# The Influence of Planting Periods on Herbivore and Natural Enemy Abundance on Yellow Sticky Traps in Bt Maize Fields

**DOI:** 10.3390/insects13040388

**Published:** 2022-04-14

**Authors:** Gemma Clemente Orta, Hugo Alejandro Álvarez, Filipe Madeira, Ramon Albajes

**Affiliations:** 1 AGROTECNIO Center, Department of Crop and Forest Sciences, University of Lleida, Rovira Roure 191, 25198 Lleida, Spain; fmadeira@morecolab.pt (F.M.); ramon.albajes@irta.cat (R.A.); 2 Department of Zoology, University of Granada, 18071 Granada, Spain; hugoalvarez01@gmail.com

**Keywords:** crop-rotation programs, integrated pest management, crop phenology, planting periods

## Abstract

**Simple Summary:**

Previous research has shown that both landscape and field variables significantly influence the abundance of herbivorous insects and their natural enemies in Bt maize; however, crop phenology was found to have the strongest effects. Therefore, here, we studied how the planting period affected the insect dynamics in Bt maize. Our data provide clear evidence that the abundance of herbivores and natural enemies peaks in earlier growth stages and that their abundance varied between maize phenology stages, but interestingly, it did not show a strong effect when the planting period changed.

**Abstract:**

Knowledge of the insect densities during crop development is necessary for adopting appropriate measures for the control of insect pests and minimizing yield losses. Within integrated pest management programs, crop rotation has been carried out in recent years, but this current trend delays the planting period for Bt maize. The small amount of available information regarding the influence of sowing Bt maize early or late on the seasonal abundance of herbivores prompted us to study these aspects in two current common planting periods in northeastern Spain in 52 maize fields over three consecutive years. We sampled the fields planted on different dates with sticky yellow traps. Our results show that only the abundances of herbivore thrips, other than *Frankliniella occidentalis*, and Syrphidae were significantly different between the two planting periods. Moreover, when we performed yearly analyses, we found significant effects of the planting period on Coccinellidae and Chrysopidae in 2015 and on *Aeolothrips* sp. in 2016 and 2017. In most of the taxa, the abundance peaks in earlier growth stages, which is related to pollination (before or during). Only the abundances of *Stethorus punctillum* and Syrphidae peak later in the season. In addition, *F. occidentalis*, aphids, Syrphidae, and Coccinellidae registered higher abundance in fields sown in the late planting period. These results highlight the effects of sowing in different planting periods on insect dynamics in Bt maize and can be used to identify the abundance of certain pests and natural enemies in specific phenological stages of maize, which may allow producers to adopt better-integrated management and thus avoid reaching the level of economic damage.

## 1. Introduction

Maize is the most common arable crop in summer in the Ebro Basin (northeastern Spain). Farmers traditionally plant maize rather early in spring, after the winter fallow, to increase its yield and ensure an optimal low grain humidity at harvest [1]. In recent years, however, due to the increasing irrigated area of agricultural land, the longer growing season caused by warmer conditions, and the decreasing revenue received by cereal growers, the alternative rotation of winter and summer cereals is intensifying. As a consequence, maize is increasingly planted after the cereal harvest during the spring, in comparison with the previously common habit of planting after the winter fallow at the beginning of spring. Therefore, maize can be planted from the end of March to early July and harvested for grain or forage from the end of September to November or even in early December.

Therefore, in the same area, maize fields with different phenologies coexist side by side; this allows herbivores and their natural enemies (NEs) to move throughout the landscape and select the maize plants of preferred age for feeding and reproduction. Many studies have determined optimal planting dates and maize cultivar cycles for maximizing yields [2,3,4], but studies have rarely addressed the influence of maize planting periods on insect pests or viral diseases. Knowledge of how planting periods may affect the composition and population dynamics of maize herbivores and their NEs could contribute to the understanding of altering or modifying agricultural practices for maize.

Herbivorous insects affect crop yield due to both their plant feeding and their virus transmission capacity. Among the herbivores feeding on maize, borers (Lepidoptera) and homopterans (Hemiptera) are the most damaging pests in the region [5]. The maize borers in the Mediterranean area belong to two species: *Sesamia nonagrioides* Lef. (Lepidoptera: Noctuidae) and *Ostrinia nubilalis* (Hbn) (Lep.: Pyralidae). Chemicals are rarely applied to control these species because of their poor efficacy due to the endophytic habits of borer larvae, the difficulty of application due to crop height, and their strong impact on NEs. Insect-resistant cultivars, particularly genetically modified maize (e.g., Bt maize), have been the most successful control method in recent years.

Other damaging insects on maize in the area include Homopterans, both Auchenorrhyncha and Sternorrhyncha [6]. Among Auchenorrhyncha, the planthopper *Laodelphax striatellus* (Fallén) (Hemiptera: Delphacidae) is harmful to maize mainly due to its capacity to transmit maize rough dwarf virus (MRDV) [7,8], and the leafhopper *Zyginidia scutellaris* Herrich-Schäffer (Hemiptera: Cicadellidae) causes a reduction in plant vigor due to its feeding on the phloem (mostly in early plant growth stages). The main group of Sternorrhyncha affecting maize yield in the area includes several species of aphids (Hemiptera: Aphididae), which, in general, are more abundant before anthesis (though this depends on the species). Their main damage comes from their high capacity to transmit two common maize viruses, maize dwarf mosaic virus (MDMV) and sugarcane mosaic virus (SCMV) [9], the two main potyviruses in this area. Although soil insecticides may cause significant homopteran suppression, they are only partially effective in preventing infections by aphid-transmitted viruses due to their nonpermanent transmission. By contrast, insecticide treatments on maize showed negative effects on NE abundance.

Clemente-Orta et al. [10] investigated the influence of the surrounding landscape and crop fields on the abundance of maize herbivore insects and their NEs in northeastern Spain. They showed that the variable with the highest effect on insect abundance was the maize growth stage. Specifically, in spring, the crop phenology was positively related to the abundance of the predators *Orius* sp. and *Propylea quatuordecimpunctata*, while *Stethorus* spp. and Syrphidae were negatively related. Conversely, in summer, the phenology was positively related to *Stethorus* spp., whereas negative relationships were found for *P. quatuordecimpunctata* and *Aeolothrips* sp. In addition, phytophagous Thripidae (except *Frankliniella* sp.), *Empoasca vitis*, aphids, phytophagous Thripidae, *Z. scutellaris,* and *L. striatellus* showed negative relationships with the crop phenology in both spring and summer. Further studies conducted by the same research group found that early and late maize planting periods had important effects on the incidence of SCMV, MDMV [11], and MRDV [12].

Following the above findings [10,11,12], we investigated the influence of two common planting dates for Bt maize in northeastern Spain on the seasonal abundance of herbivores and their NEs. We tested the hypothesis that the planting period for maize could have a greater effect on the abundance of insects than the maize phenology or interannual variation. We addressed this by sampling 52 maize fields sowed with early (March–April) and late (May–April) planting periods for three consecutive years.

## 2. Materials and Methods

### 2.1. Study Area and Cultivation Practices

**Figure 1:**
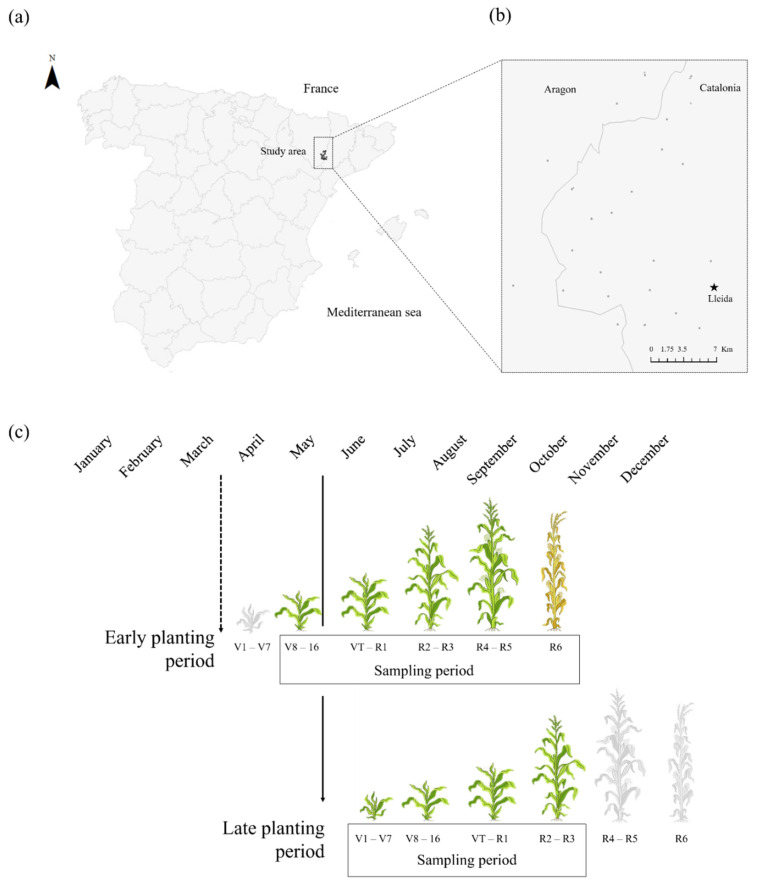
Maize-growing areas in Ebro valley, northern Spain: (**a**) study area, (**b**) the maize fields sampled, and (**c**) the two common planting periods in the area (early and late) with the maize phenology.

This study was carried out in 2015, 2016, and 2017 in the Ebro Basin (41°48′12.20″ N, 0°32′45.77″ E; 120–346 m altitude; 200–400 mm rainfall, Tmin: 8–24 °C and Tmax: 18–38 °C) (Figure 1a). In the region, the most traditional crop rotation included winter and summer cereals and alfalfa. The pest management for the cereals includes pre- and post-emergence herbicide applications. During the study period, soil treatments with neonicotinoids were still allowed, and this was a general practice in the area together with routine seed treatment with fungicides. The maize variety used in the region is Bt maize (hereafter simply called maize). Under these conditions, a total of 52 maize fields were selected to have a wide variety of planting dates so that several growth stages coexisted throughout the season in the area (Figure 1b). The sizes of the fields varied between 0.9 and 13.68 ha, and the fields were located at least 2 km apart from each other. The growth stage intervals of the maize plants were recorded using the Ritchie et al. [13] nomenclature. For analysis, the fields were grouped into two groups: The early planting period included those sown from March until the end of April, whereas late-planted fields were those planted during May or June (Figure 1c). The fields sampled were 6 fields in 2015 (3 early vs. 3 late sown fields), 23 fields in 2016 (14 early vs. 9 late sown fields), and 23 fields in 2017 (13 early vs. 10 late sown fields).

### 2.2. Sampling of Herbivores and NEs

The abundance of insects in the maize fields was estimated using yellow sticky traps (30 × 25 cm, Pherocon Trece, Adair, OK, USA). In each field, 3 traps were placed at crop canopy height along a transect perpendicular to the nearest edge (attached on stakes approximately 30 m apart), and the traps were separated from each other by 15 m [10]. Sampling was carried out on the following date (sampling date, SaD) intervals: SaD1: 15 May–15 June; SaD2: 22 June–10 July; SaD3: 10–22 August; SaD4: 31 August–20 September. The traps were left active for 1 week; then, the traps were collected and stored at 6–8 °C until insect identification. The insects were identified at different taxonomic levels: family, genus, and/or species. The crop growth stages were grouped into 6 intervals: (1) V1–V7; (2) V8–V16; (3) VT–R1; (4) R2–R3; (5) R4–R5; (6) R6.

### 2.3. Statistical Analysis

We fitted generalized linear mixed models (GLMMs) to analyze the insect population dynamics (as repeated measures), for which we used the weekly mean numbers of the three traps placed in each field (i.e., the mean among the three traps in each week) as the dependent variable in all the models. We used the negative binomial tendency. Firstly, to compare the effect of the planting period on insect abundance for all the years (overall effect), we included two planting periods (early and late), maize phenology, and their interaction as fixed effects, and the site identification (ID) and year were included as random effects (glmer.nb (insect_abundance ~ planting × phenology + (1|site) + (1|year), data = data)).

Secondly, to compare the effect of the planting period on insect abundance for each year, we included the same variables in a model per year (glmer.nb (insect_abundance ~ planting × phenology + (1|site), data = data)). Thirdly, to identify the interannual variation (temporal effect), we included the year as a fixed effect and maize phenology and site ID as random effects (glmer.nb (insect_abundance ~ year + (1|phenology) + (1|site), data = data)). Further differences between the groups in each model were tested using contrast post hoc tests. All analyses were conducted in the R software v 3.6.2 [14]. For each model, we tested for significant differences (5%; *p* < 0.05) using the package car. GLMMs, and post hoc tests were performed using the lme4 and multcomp packages, respectively.

## 3. Results

A total of 316,564 insects were trapped in 585 yellow sticky traps placed in the 52 maize fields during the three years of study. The herbivorous insects identified included *Frankliniella occidentalis*, “other herbivore thrips” (others different from *Frankliniella* sp.), *Zyginidia scutellaris*, *Laodelphax striatellus*, *Empoasca* sp., and aphids. The NEs identified were *Orius* sp., *Nabis* sp., Miridae, *Stethorus punctillum*, *Chrysoperla* sp., Syrphidae, *Aeolothrips* sp., and Coccinellidae. The total numbers of insects trapped per year were 201,775 in 2016 (*n* = 23 fields), 75,250 in 2017 (*n* = 23), and 39,539 in 2015 (*n* = 6).

In contrast to what was initially hypothesized, the planting period had no significant effect on insect numbers for any of the taxa recorded except for the group of “other herbivore thrips” (Table 1), in which fields that were planted earlier showed higher numbers. However, when this factor interacted with phenology, five taxa exhibited significant results (Table 1 and Table 2). When the influence of the planting period was analyzed within each of the three years, of the 36 possibilities (12 insect taxa per 3 years), we only found significant differences (i.e., differences between planting periods) in three taxa, with higher values in the early planted fields: in 2015 for Coccinellidae (*χ*^2^ = 6.76; *df* = 1; *p* = 0.0002) and Chrysopidae (*χ*^2^ = 7.94; *df* = 1; *p* = 0.004), and for *Aeolothrips* sp. in both 2016 (*χ*^2^ = 5.02; *df* = 1; *p* = 0.02) and 2017 (*χ*^2^ = 11.7; *df* = 1; *p* = 0.006).

**Figure 2:**
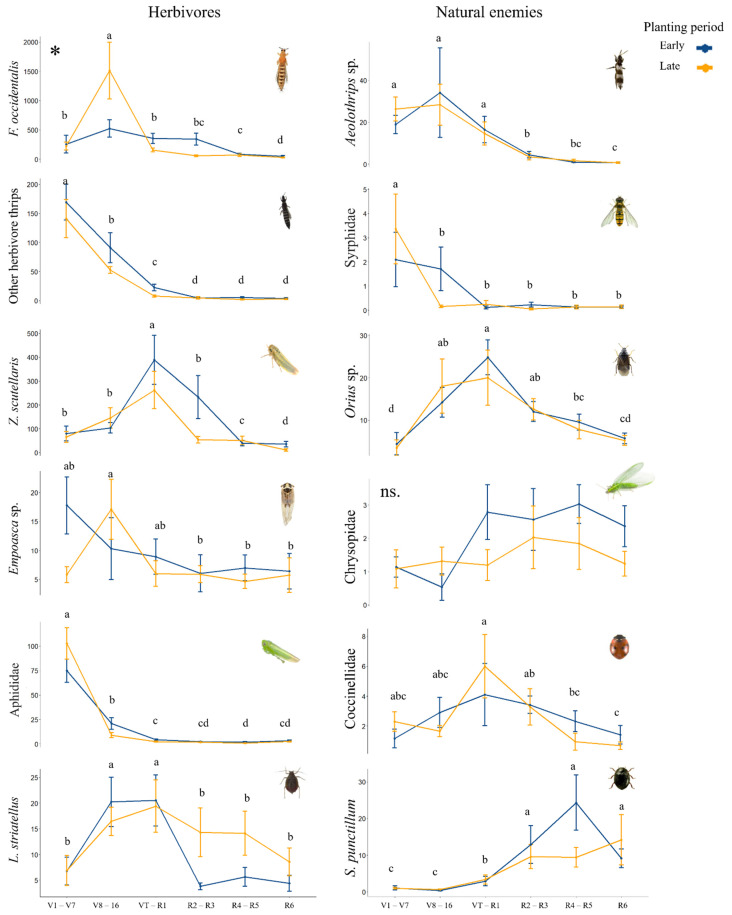
Insect abundance according to maize phenology for all years in each planting period (early and late) with the mean and standard deviation. The letters show significant differences (*p* < 0.05) among phenologies. Asterisks indicate that there are differences between planting periods. Chrysopidae show non-significant (ns) results. (More details in Supplementary Material).

The maize phenology (indicated by the growth stage intervals at the sampling week) had significant effects on the abundance of most of the NEs (except Chrysopidae) (Table 2) and all the herbivores (Table 1) when analyzed alone or interacting with the planting period. In particular, the number of insects varied with the crop growth stage, with clear peaks in certain growth stages (Figure 2). The abundance of most of the herbivores and NEs peaked before or at pollination in both early and late-planted fields. The “other herbivore thrips”, together with aphids and Syrphidae, showed their highest abundance in the earliest growth stage (V1–V7), whereas the genus *Empoasca* sp. peaked in the first and second growth stage intervals in fields planted early and late, respectively. The herbivore *F. occidentalis* showed only a high peak in fields planted late, which occurred in the second growth stage interval, whereas the rest of their numbers were low throughout the entire season, independent of the planting period. The predator *Aeolothrips* sp. peaked at the second growth stage interval in both early and late-planted fields. The rest of the herbivores and NEs peaked close to the pollination growth stage and independent of the planting period; only Chrysopidae, especially *S. punctillum*, peaked after pollination and reached significantly higher numbers in R4–R5.

The second type of analysis was performed to observe the interannual variation in insect abundance along the season per year. Overall, the dynamics of insects during the season were quite consistent in the three years for most of the taxa recorded. Moreover, the insect prevalence was quite similar in the three years for both herbivores and NEs.

**Figure 3:**
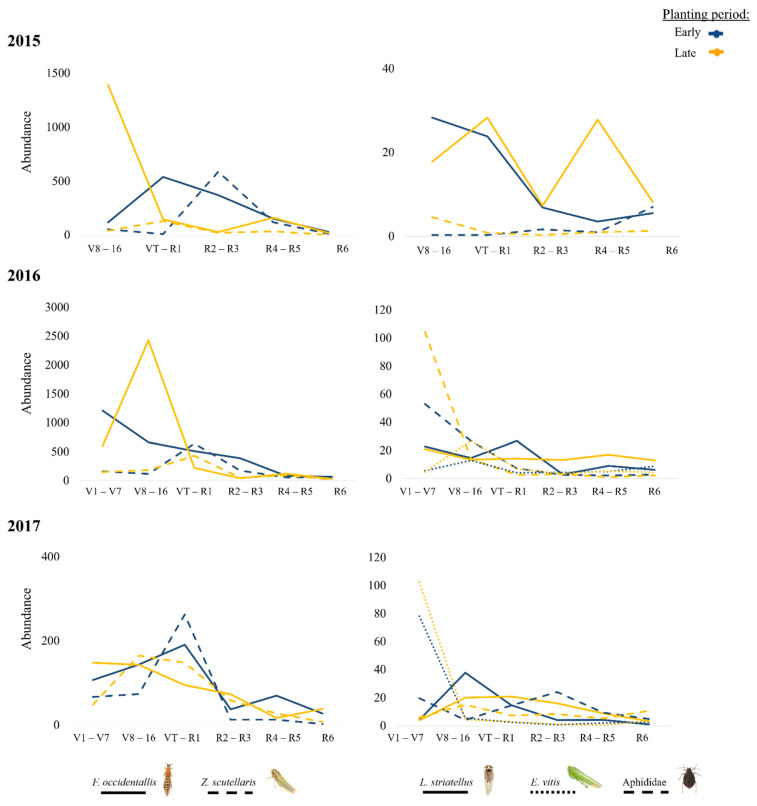
Herbivore abundance according to the maize phenology each year in each planting period (blue line = late; yellow line = early). The species that had significant differences (*p* < 0.05) in the analyses are shown. (More details in Supplementary Material).

**Figure 4:**
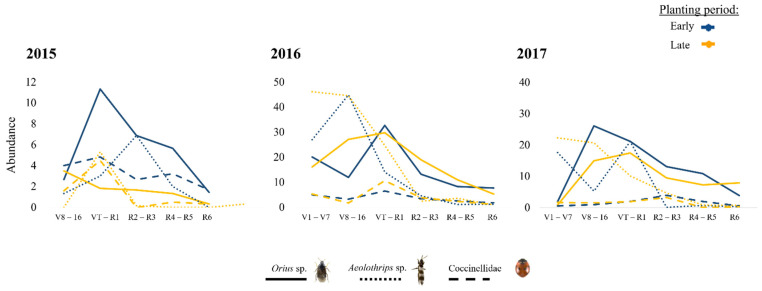
Natural enemy abundance according to the maize phenology each year in each planting period (blue line = late; yellow line = early). Only the species that had significant differences (*p* < 0.05) according to the analyses are shown. (More details in Supplementary Material).

Among herbivores, the highest numbers were recorded for *F. occidentalis*, followed by *Z. scutellaris*. Among NEs, *Orius* sp. was the most abundant taxon, followed by *Aeolothrips* sp. and *S. punctillum*. The analysis of interannual variation also showed that the abundance of insects collected in early vs. late planting periods followed a rather similar pattern, although it varied significantly in the cases of five herbivores—*F. occidentallis*, *Z. scutellaris*, *L. striatellus*, *E. vitis*, and Aphididae (Figure 3)—and three NEs, i.e., *Orius* sp., *Aeolothrips* sp., and Coccinellidae (Figure 4).

## 4. Discussion

The diversity and abundance of the maize insect taxa collected on the yellow sticky traps were similar to the ranges reported by other authors in previous studies conducted in the region over the last 15 years with the same traps or visual on-plant sampling [6]. Among the herbivores, thrips and hemipterans were predominant, while among the predators, the generalist *Orius* sp., the *Aeolothrips* sp., and the mite-feeding specialist *S. punctillum* were the most abundant taxa recorded. While we initially expected that the planting period would have a strong effect on the total insect abundance of the crop, when we analyzed this factor, we did not find remarkable effects in the different models for most of the taxa recorded. This result could indicate that the damage caused by herbivores to the crop is not strongly influenced by the maize planting period. The maize phenology was the most influential factor for insect abundance. Thus, differences in insect abundance according to the planting period were found at particular growth stages, which is significant in terms of the potential crop damage caused for maize, as maize plants have different susceptibilities and yield responses to herbivores and disease vectors; this is important during the initial vegetative stages [15] because phytophagous insects might prefer softer leaves (such as those of the initial stages of the plant in other cereals) [16].

Population peaks in the early growing stage (before the reproductive stages) were common for the herbivorous insects, as others have shown [17,18,19,20,21]. The population peak of *Empoasca* sp. was different between the planting periods, but in fields sown at earlier dates, the peak was in the V1–V7 stage, while in late fields, it was in a more advanced vegetative stage, V8–V16. However, *Z. scutellaris* showed the greatest abundance in the pollination stage in both planting periods. Some studies have also shown a variation in Cicadellidae’s population peaks. Thus, in contrast to the study of Ribeiro et al. [19], in Brazil, which found that the population peaks of three Cicadellidae species were in the initial vegetative stages of maize, V1–V3, in a study conducted in Argentina with several Cicadellidae species, the highest abundance was found between stages V6 and V8 [20]. In a study by Bastos et al. [21], there were the highest populations of Cicadellidae in the reproductive phase of maize. In all the referenced studies, however, population peaks were recorded before or just at reproductive stages independently of the planting period, which is consistent with the results of the present study.

The population peak of the aphids was particularly observed in V1–V7, which is important for the vectors of maize potyviruses [9,10]. Aphids that are able to transmit maize potyviruses were not found to be influenced in their abundance by the planting period at any growth stage in the present study. In [22], Maia et al. studied the effect of the phenological stage of maize on the aphid infestation and concluded that there was a higher incidence of aphid colonies in the V4 stage, which has been recorded in other cereals [23]. In the case of MRDV, its vector, *L. striatellus*, did show significantly higher abundances in late-planted fields but only at late growth stages; in this phenological stage, virus transmission is already complete [7,8,11] and the number of vectors is rather irrelevant when considering the virus incidence. For other herbivores, *F. occidentalis* was approximately three times more prevalent in fields planted later than early, but in both cases, this happens in the same stage, V8–V16, while “other herbivore thrips” were more abundant in V1–V7. Most of the information on this issue classically refers to the role of the secondary metabolite 2,4-dihydroxy-7-methoxy-1,4-benzoxazin-3-one (DIMBOA), which is a hydroxamic acid found in Poaceae plants, such as maize, that acts as a direct defense against herbivores [24], maize borers [23,25], and aphids [26]. However, as DIMBOA and its related compounds decrease with plant age [27], it does not seem that its role in shaping the abundance of insect herbivores is crucial, and other causes of insect decline after maize pollination should be investigated to understand the herbivore insect preference for particular maize growth stages.

Most of the NEs recorded in this study peaked soon after the herbivores did, as in the case of *Aeolothrips* sp. and *F. occidentalis*, “other herbivore thrips”, and *Orius* sp. and hemipterans, which is a phenomenon already noted in maize by Albajes et al. [28] and Ardanuy et al. [29]. Only *S. punctillum* peaked in late phenological stages, R4–R5, probably soon after red spider mite populations peak. For other NEs, such as lady beetles, young maize stages may act as refugia and attract predator individuals in the absence of suitable prey due to favorable microclimate conditions as those found by Pan et al. [30].

Finally, although the study focused on the influence of the planting period on the abundance of insects, other factors such as pesticide treatments could also have an influence on the number of insects. However, this was not the case in our study; only pyrethroid insecticides were applied in the soil of most of the fields included in this study, to prevent damage from wireworms and soil worms. These insecticides have no systemic activity, and it can be assumed that these treatments had no influence on the abundance of aerial insects. As the insecticide treatments were common for most of the fields in the pre- and post-emergence periods and did not interfere with maize growth or aerial insect incidence, they did not need to be accounted for in the statistical models. In addition, the composition and configuration of the landscape could modulate the influence of field planting periods on herbivores and NE insects [10] as a consequence of insect movement among habitats, which results in spatial or temporal migrations [31]. Thus, the combination of many trophic-level interactions, the landscape structure, the management of crop fields, warmer conditions, and the changes in agricultural policy (management programs, the cereal market, or maize prices) make it difficult to fully understand and predict the changing patterns of insect abundance in particular agricultural habitats.

## 5. Conclusions

A number of potential consequences derived from varying maize planting periods can be expected. The pressure of insect pests on maize and the total number of NEs did not change substantially according to the planting period. Most of the consequences will probably come from the different susceptibilities of maize plants of different ages to insects and insect–virus vectors. Most of the insect populations studied in this study peaked before or during maize plant pollination. In this period, high differences in plant phenology may lead to yield reduction. In parallel, although the number of generalist predators was not greatly influenced by the differential amount of prey (herbivores), according to the planting period, the specialist Nes may indeed be influenced by it. Finally, considerations about the landscape structure, as well as field and crop management practices, may also modulate the impact of the maize planting date on insect dynamics; therefore, further studies on the influence of the planting date on insect herbivores and their Nes should include both factors.

## Figures and Tables

**Table 1:** Results of the generalized linear mixed models of herbivore abundance according to the maize phenology and planting period (early and late) and their interaction for all years.

Herbivore	Variable	Chisq	Df	*p* Value
*F. occidentalis*	Planting	1.437	1	0.23
	Phenology	147.29	5	**<0.001**
	Planting:Phenology	210.84	5	**<0.001**
“Other herbivore thrips”	Planting	12.62	1	**<0.001**
	Phenology	494.35	5	**<0.001**
*Z. scutellaris*	Planting	0.77	1	0.38
	Phenology	160.92	5	**<0.001**
*Empoasca* sp.	Planting	0.201	1	0.65
	Phenology	18.08	5	**0.002**
	Planting:Phenology	11.98	5	**0.03**
Aphids	Planting	3.31	1	0.06
	Phenology	564.93	5	**<0.001**
*L. striatellus*	Planting	0.42	1	0.51
	Phenology	66.36	5	**<0.001**
	Planting:Phenology	15.3	5	**0.009**

**Table 2:** Results of the generalized linear mixed models of NE abundance according to the maize phenology and planting period (early and late) and their interaction for all years.

Natural Enemy	Variable	Chisq	Df	*p* Value
*Aeolothrips* sp.	Planting	0.01	1	0.89
	Phenology	207.51	5	**<0.001**
Syrphidae	Planting	0.50	1	0.47
	Phenology	79.73	5	**<0.001**
	Planting:Phenology	14.72	5	**0.01**
*Orius* spp.	Planting	2.3	1	0.12
	Phenology	82.98	5	**<0.001**
Chrysopidae	Planting	1.97	1	0.16
	Phenology	10.28	5	0.06
Coccinellidae	Planting	1.27	1	0.25
	Phenology	25.63	5	**<0.001**
*S. punctillum*	Planting	0.22	1	0.63
	Phenology	134.75	5	**<0.001**

## Data Availability

The data presented in this study are openly available in FigShare at https://doi.org/10.6084/m9.figshare.19565929.v1.

## Supplementary Materials

The following supporting information can be downloaded at: https://www.mdpi.com/article/10.3390/insects13040388/s1, File S1: Extra model results regarding Figure 2, Figure 3 and Figure 4.
