# Supplementary material for: The Influence of Planting Periods on Herbivore and Natural Enemy Abundance on Yellow Sticky Traps in Bt Maize Fields
Source: Insects. 2022 Apr 14;13(4):388. doi: 10.3390/insects13040388 (PMC9030327; doi:10.3390/insects13040388)
Supplement: Supplementary file 1 [file insects-13-00388-s001.zip › insects-1620933-supplementary.pdf]

Supplementary material: The influence of planting periods on herbivore and natural enemy abundance on yellow sticky traps in Bt maize fields. Clemente-Orta et. al

S1. Data

año	Site	Pheno	Sowing	F. occiden	Other thrips	Zyg	Laodelphax	Empo	Aphid	Orius	Steth	Crisopa	Aeolothrips	Sirf	coc
2017	Alcarras	V1-V7	Late	43,3333333	52,6666667	182	3,33333333	2,66666667	196,333333	0	1	0	9,33333333	0,33333333	1
2017	Bassanova1	V1-V7	Late	10,6666667	218	31	0,66666667	6,33333333	144,666667	0,66666667	2,33333333	0,66666667	4	1,33333333	2
2017	Ramon Vidal	V1-V7	Early	7,33333333	445	157,333333	6,33333333	39,6666667	79	0,33333333	0,33333333	0	3,33333333	0,66666667	0
2017	Alcarras-Gim1	V1-V7	Late	231,666667	101,666667	4,66666667	2,66666667	6,33333333	74,6666667	0	1,66666667	0	27,3333333	4	1,33333333
2017	Manoleta	V1-V7	Late	317,666667	169	165	25,3333333	7	81,6666667	1,66666667	1	0	30,6666667	1,66666667	2,33333333
2017	Circuito	V1-V7	Early	27,3333333	107	16,3333333	0,33333333	2,66666667	61,6666667	0	2	0,66666667	7,33333333	0,66666667	0
2017	Miguel Velo1	V1-V7	Early	45	122,666667	5,33333333	0	27,6666667	39,6666667	0,33333333	1,5	2	33,6666667	0,33333333	1,33333333
2017	Bassanova2	V1-V7	Early	9	103,666667	16	0	0,66666667	59	0,33333333	0,33333333	0	2,66666667	0,33333333	0,33333333
2017	Altorrico2	V1-V7	Late	114	159,333333	12,6666667	3,66666667	4,66666667	18,6666667	1,66666667	1,33333333	3	18,6666667	14,3333333	2,33333333
2017	Canal	V1-V7	Early	11,3333333	43,3333333	436,333333	1	3,33333333	39,3333333	0	0,33333333	0,33333333	6,66666667	0	0
2017	Jardiland	V1-V7	Late	117	30	26,6666667	1	2,66666667	193	1,66666667	0	0	21	1	0,33333333
2017	Diarte	V1-V7	Early	31,6666667	125	28,6666667	0	9,66666667	89,6666667	0	0,33333333	1,33333333	15,6666667	0	1
2017	Pantano	V1-V7	Late	25,3333333	128,666667	26,6666667	0,33333333	20	61	0	1,33333333	1	43,3333333	0	1,33333333
2017	Altorrico1	V1-V7	Early	77,6666667	132,666667	8	1,66666667	50,6666667	103,666667	0,33333333	0	0	2	2	0
2017	Josep Tomás	V1-V7	Late	187,333333	451	2,66666667	0	2	99	1	2	0	3,66666667	0	0
2017	Gimenells	V1-V7	Late	7,66666667	58	9,33333333	0	3,66666667	77,3333333	0	0,66666667	1,33333333	2,33333333	1,33333333	1
2017	Alcarras-Gim2	V1-V7	Early	527	125,666667	92,6666667	16	3,33333333	51	2,66666667	0	3,33333333	29,3333333	17,3333333	1,33333333
2017	Carrasumada1	V1-V7	Early	386,333333	38	52,6666667	29	12,6666667	29	20,6666667	0	1,33333333	44,6666667	0	0,66666667
2017	Aeropuerto	V1-V7	Late	429	156	22,3333333	3,66666667	4,66666667	77,6666667	2,33333333	0,33333333	6,66666667	62,6666667	13	5,33333333
2017	Sucs	V1-V7	Early	8,33333333	295	6,33333333	1	1,66666667	71,3333333	0,33333333	0,33333333	0	16,3333333	0	1,33333333
2017	Mirador	V1-V7	Early	75,3333333	250	20	0	46,3333333	110	0	0,66666667	2,66666667	16	0,66666667	1
2017	Vencillo	V1-V7	Early	145,666667	227,333333	6,33333333	0,33333333	48,6666667	66,6666667	0	8,33333333	3	10,6666667	1,66666667	0,66666667
2017	Nyaco	V1-V7	Early	40	145	22,6666667	0,33333333	8,66666667	216	0,33333333	1,66666667	0,66666667	41	4	0
2017	Pantano	VT-R1	Late	218	11	113,666667	45	4,33333333	5,66666667	38,6666667	0,33333333	5	19	0	7
2017	Vencillo	VT-R1	Early	315,333333	45,6666667	122	7,66666667	4	2,66666667	25	0,33333333	6,33333333	28,3333333	0	0,33333333
2017	Mirador	V8-V16	Early	353,333333	16	48,3333333	14	2,33333333	5	48,6666667	0	0,33333333	3	0	0

2017	Canal	VT-R1	Early	79,6666667	3,33333333	182,666667	18,3333333	0,66666667	0,66666667	17,6666667	0	0,33333333	1,33333333	0	6
2017	Josep Tomás	V8-V16	Late	148,666667	44	15	11,3333333	8,66666667	1	3,33333333	0	5,66666667	38	0	3
2017	Bassanova2	VT-R1	Early	87,6666667	4,33333333	170,666667	7,66666667	3,33333333	2	17,3333333	0	1	17	0,33333333	0,33333333
2017	Diarte	VT-R1	Early	125,333333	0	20,6666667	4	0,66666667	0,66666667	1	0	0	2	0	4,33333333
2017	Miguel Velo1	VT-R1	Early	265,666667	4,33333333	106,333333	13,6666667	20,6666667	2,66666667	41,6666667	1,33333333	2	116,666667	0	3
2017	Ramon Vidal	VT-R1	Early	54	41,6666667	399,333333	21,3333333	1	4,66666667	40	0,66666667	1,33333333	0,33333333	0	0,33333333
2017	Bassanova1	V8-V16	Late	53,3333333	73,3333333	72	30,3333333	0,33333333	3	8,33333333	0,66666667	0	0,66666667	0	1,33333333
2017	Nyaco	VT-R1	Early	565	62,6666667	225,333333	11,6666667	25,6666667	2,66666667	17,3333333	0	1,66666667	22,3333333	0,66666667	0
2017	Gimenells	V8-V16	Late	187,333333	98,6666667	11	1,66666667	29,3333333	2,33333333	10,3333333	0	3,66666667	16,3333333	0	1
2017	Altorrico1	V8-V16	Early	40	38	63	17,3333333	1,66666667	1,66666667	18	0	0	6,33333333	0	2,66666667
2017	Carrasumada1	VT-R1	Early	59	5,33333333	1306,66667	11	38,6666667	0,33333333	36	0,33333333	0,66666667	7,33333333	0	0
2017	Alcarras	V8-V16	Late	191	40,3333333	687,333333	23	24,6666667	1,66666667	37,6666667	0,66666667	0	19,6666667	0	2,33333333
2017	Manoleta	V8-V16	Late	138	80,3333333	41	34	12,3333333	14,3333333	15,3333333	0,66666667	0	29	0	0,33333333
2017	Circuito	V8-V16	Early	38,3333333	34	110	82,3333333	8,33333333	10	11,6666667	0	0,33333333	6,66666667	0	0,33333333
2017	Sucs	VT-R1	Early	300,666667	15,3333333	29	9,33333333	2,33333333	3,66666667	5,66666667	0	10,3333333	16	0	5
2017	Alcarras-Gim2	VT-R1	Early	60	18,3333333	65	43	47,6666667	6,33333333	10,3333333	7	2	0	0	0,66666667
2017	Altorrico2	R2-R3	Late	115,666667	32,6666667	90	59,3333333	18,3333333	2,66666667	4,66666667	1,33333333	0	9,66666667	0	4,66666667
2017	Jardiland	VT-R1	Late	42,6666667	15,6666667	339,666667	46,3333333	20,3333333	4,66666667	17,3333333	3	1,33333333	6,33333333	0	0
2017	Aeropuerto	VT-R1	Late	179,666667	0	285,666667	9	2,66666667	0	11	13	0	25,3333333	0,33333333	2,66666667
2017	Alcarras-Gim1	R2-R3	Late	85,6666667	0,66666667	96,3333333	27,6666667	8,66666667	0,66666667	4	14,3333333	0,66666667	2,66666667	0	3
2017	Gimenells	R4-R5	Late	46	1,33333333	105,666667	3	5,66666667	0	7,66666667	20	9	3,66666667	1	0
2017	Circuito	R4-R5	Early	31,3333333	2,66666667	4,33333333	4,33333333	3,33333333	0,33333333	21	41,3333333	4	0,33333333	0	2,33333333
2017	Bassanova2	R4-R5	Early	41,3333333	10,3333333	39,6666667	1,33333333	4	1	9,66666667	19,3333333	3	0,66666667	0	0,66666667
2017	Bassanova1	R2-R3	Late	29,6666667	3	33,3333333	5,66666667	3,66666667	0,33333333	17,3333333	15	12,6666667	0,66666667	0	2,33333333
2017	Josep Tomás	R2-R3	Late	107,333333	1,66666667	169,333333	1,33333333	14,6666667	0	15,6666667	22	3,33333333	18,6666667	0	1,33333333
2017	Alcarras	R4-R5	Late	6,66666667	2,33333333	71,3333333	8,66666667	7,33333333	0,66666667	10,3333333	32,6666667	2,66666667	0	0	0,66666667
2017	Carrasumada1	R4-R5	Early	56	29,3333333	45,3333333	7,66666667	9	1,66666667	19,3333333	29,6666667	6,33333333	0,66666667	0	0,33333333
2017	Diarte	R4-R5	Early	22,6666667	2	16	5,66666667	0	0,33333333	4,66666667	5	0,66666667	0	0	0,33333333

2017	Altorrico1	R4-R5	Early	53,6666667	2	6,33333333	4,66666667	3	1,33333333	11,6666667	8,33333333	0,33333333	1	0	8,66666667
2017	Manoleta	R2-R3	Late	172	3,66666667	16,6666667	18,6666667	6,33333333	3,33333333	9	5,33333333	2,33333333	1,33333333	0,33333333	11,6666667
2017	Vencillo	R4-R5	Early	175,666667	11,6666667	9,33333333	1	1,66666667	2	2	74	4	0,33333333	0	1,33333333
2017	Miguel Velo1	R2-R3	Early	62,6666667	9,66666667	18,6666667	5,66666667	45,6666667	1,66666667	16	64,3333333	7,66666667	0,33333333	0,33333333	5,66666667
2017	Mirador	R4-R5	Early	95,3333333	10,3333333	9,33333333	5,33333333	4	0,33333333	10,3333333	16,6666667	12,3333333	0,33333333	0,33333333	2
2017	Canal	R2-R3	Early	12,6666667	1	7,33333333	2,66666667	2,66666667	0	10,3333333	3,66666667	0	0	0	2,33333333
2017	Ramon Vidal	R4-R5	Early	168	7,33333333	18,6666667	2,66666667	2,33333333	0,66666667	7,66666667	102	1	0,66666667	0,33333333	6
2017	Nyaco	R4-R5	Early	38	4	16,3333333	1	2,33333333	0,33333333	12,3333333	50,6666667	2	0	0,33333333	0
2017	Sucs	R4-R5	Early	206,333333	5	8,66666667	5,33333333	0,66666667	0,33333333	9,33333333	32,3333333	4,66666667	3	0	7,33333333
2017	Alcarras-Gim1	R4-R5	Late	16	0	2	4,33333333	5,66666667	0,66666667	8	0	0	0	0	0
2017	Alcarras-Gim2	R4-R5	Early	13	3,33333333	1,33333333	2	15,6666667	8,33333333	8,66666667	0,33333333	0	0	0,33333333	0
2017	Gimenells	R2-R3	Late	4,66666667	0	6	0	3,33333333	0,66666667	11,3333333	1	1	0	0	0
2017	Aeropuerto	R2-R3	Late	1	0,33333333	0	0	4	0,66666667	4,66666667	0,33333333	0	0	0	0
2017	Altorrico2	VT-R1	Late	18,3333333	7,33333333	2	1,33333333	8	2,33333333	6,33333333	1,66666667	0	0	0	0
2017	Jardiland	VT-R1	Late	17,6666667	3,66666667	1,33333333	3	1,66666667	1,66666667	14	1,33333333	0	0,33333333	0	0
2017	Gimenells	R4-R5	Late	2,33333333	0,33333333	26,6666667	1	2,66666667	1	3	19	9	0	0	0
2017	Circuito	R4-R5	Early	10,3333333	0	6,33333333	0,66666667	1,33333333	1	4,33333333	2,66666667	0	0	0	0,66666667
2017	Miguel Velo1	R4-R5	Early	27,3333333	1	1,66666667	1,66666667	36	3	20,3333333	4,66666667	5	0	0	0
2017	Manoleta	R6	Late	33,6666667	0	1,66666667	11,6666667	5,66666667	5,33333333	10,6666667	1,33333333	2	0	0	0,33333333
2017	Carrasumada1	R6	Early	68,3333333	1,66666667	2,33333333	2	8	3	4,66666667	0,66666667	5,33333333	0,33333333	0,33333333	0
2017	Ramon Vidal	R6	Early	57,3333333	1,66666667	1,66666667	0	3	2	6,33333333	8,66666667	1	0,33333333	0	0
2017	Altorrico1	R6	Early	12,6666667	1	0,66666667	1,66666667	1,33333333	1	10,3333333	8,66666667	0,33333333	0	0,33333333	4
2017	Altorrico2	R4-R5	Late	10,3333333	2	0,33333333	18,3333333	3	0,66666667	10,6666667	6,66666667	0,33333333	1	0	0,33333333
2017	Vencillo	R6	Early	38,6666667	1,33333333	0,33333333	0,33333333	3	1,33333333	0	8,33333333	0	1	0,33333333	0
2017	Jardiland	R4-R5	Late	41	3	9,33333333	20,6666667	3,66666667	1	5	1,33333333	1	2,33333333	0	0
2017	Pantano	R6	Late	123,333333	17	7	1	43,3333333	6,66666667	4	13	0	3	0	0,33333333
2017	Alcarras-Gim2	R4-R5	Early	38,6666667	4,66666667	2	21	44,6666667	6,33333333	18,6666667	3	0,66666667	1,66666667	0,66666667	0
2017	Alcarras-Gim1	R4-R5	Late	11,3333333	0,33333333	5	15,3333333	12,6666667	0,33333333	4	1	0	0	0	0

2017	Bassanova1	R6	Late	20,6666667	0,66666667	5,66666667	0	2,66666667	3	8,33333333	44,6666667	2	0,33333333	0,33333333	0,66666667
2017	Canal	R6	Early	8,33333333	0,33333333	8,66666667	0,66666667	18	3,33333333	1	3	0	0,33333333	0	0,66666667
2017	Josep Tomás	R6	Late	11,3333333	2	23	0,33333333	0,33333333	1,66666667	14,3333333	93,3333333	0	0,33333333	0	0,33333333
2017	Nyaco	R6	Early	29,3333333	6,66666667	4	0	2,33333333	8,66666667	2,33333333	19,3333333	0	1	0	0
2017	Mirador	R4-R5	Early	74,6666667	14	6,66666667	0,33333333	13,3333333	3,66666667	3,66666667	19,3333333	0,66666667	2,33333333	0,33333333	0,33333333
2017	Alcarras	R6	Late	3	0,66666667	1,33333333	4	2	0	2,33333333	1,66666667	0,66666667	0	0	0
2017	Sucs	R6	Early	4,66666667	0,33333333	2,33333333	1,66666667	2,33333333	0,66666667	3,33333333	1,66666667	1	0	0	0
2017	Diarte	R6	Early	4	0,66666667	1,66666667	2,33333333	1,66666667	0,33333333	3,33333333	1	1,33333333	0	0	0
2017	Aeropuerto	R4-R5	Late	2,66666667	0	2,33333333	0	2,33333333	0	9,66666667	9	0	0	0	0
2016	Circuito	V8-V16	Late	142,333333	28	49,3333333	12,6666667	1,66666667	22,3333333	0,66666667	0	0,33333333	9,33333333	0	0,33333333
2016	Miguel Velo1	V8-V16	Early	377,666667	34,3333333	59,6666667	11	2	29	7	1	0	15,6666667	0	4
2016	Gimenells	V8-V16	Early	85,3333333	24,6666667	43,3333333	12	3	9,66666667	14	0,33333333	0	3,33333333	0	1
2016	Vencillo	V1-V7	Early	2280,33333	341	81,6666667	26,3333333	6	29	36	0	0	52	2	9,66666667
2016	Aurora	V8-V16	Early	583,666667	137,666667	72,6666667	5	83,3333333	20,3333333	9	0	0	59,6666667	0	0,66666667
2016	Sucs	V8-V16	Early	229	115,333333	59,3333333	18,6666667	1,33333333	4,33333333	1,33333333	0	0	19	2	2,66666667
2016	Nyaco	V8-V16	Early	722,333333	338	44	23	2,66666667	62	1,66666667	0	0	11,3333333	13,6666667	4
2016	Pantano	V8-V16	Late	236,333333	53,6666667	9	11	1,33333333	42,3333333	0,66666667	0,66666667	0	36,6666667	0,33333333	2
2016	Mirador	V8-V16	Early	1552	300	287,333333	25	12,6666667	68,3333333	38	1	0,66666667	15,3333333	2,33333333	2,66666667
2016	Diarte	V8-V16	Early	578,666667	62,6666667	34,3333333	8,66666667	5	9,33333333	8	0	0	2,66666667	0,33333333	0
2016	Ramon Vidal	V8-V16	Early	1021,66667	87,6666667	169,666667	4,33333333	8,66666667	13,3333333	19,3333333	1	0,33333333	328,666667	3,33333333	2
2016	Canal	V1-V7	Early	144,666667	37	241,666667	19,3333333	5	77	4,33333333	0	1,66666667	2	1,66666667	0,33333333
2016	Manoleta	V8-V16	Early	131,666667	20,6666667	53	24	3,66666667	5	2	0	0	17	0	1,66666667
2016	Jardiland	V1-V7	Late	413,333333	45	224,666667	13,3333333	6,33333333	147,666667	21,6666667	0	0	46,6666667	0	2,66666667
2016	Altorrico2	V1-V7	Late	784,333333	122,666667	74	28,6666667	3,33333333	61	10,6666667	0	0,33333333	45,6666667	3,33333333	8
2016	Miguel Velo2	V8-V16	Early	88,3333333	70,3333333	291,666667	17,3333333	5	58,3333333	8	0,66666667	0,33333333	9,66666667	2	2
2016	Miguel Velo2	VT-R1	Early	140,333333	23,6666667	1441,33333	1,33333333	3	15,3333333	13,6666667	1,33333333	1	4,33333333	0	0,33333333
2016	Alcarras	V8-V16	Late	502,666667	73,6666667	244,333333	38,6666667	38,6666667	2,33333333	23,3333333	0,66666667	1	4,66666667	0	2,66666667
2016	Manoleta	VT-R1	Early	114,666667	8,66666667	444,666667	6,33333333	3,66666667	0,66666667	8,66666667	4	0,33333333	67,3333333	0	6

2016	Alcarras-Gim2	V8-V16	Early	1925	72,6666667	165,333333	12,3333333	14,6666667	15,3333333	23,6666667	0,33333333	6	11,6666667	2	16
2016	Nyaco	VT-R1	Early	831,666667	34	172,666667	92	2	9,33333333	37,6666667	0,66666667	0,66666667	2,33333333	0	2,33333333
2016	Alcarras-Gim1	V8-V16	Late	3390	33	254	8,33333333	23	8,33333333	13,6666667	0	0,66666667	39	0,33333333	0,33333333
2016	Altorrico1	V8-V16	Late	4225,66667	39,6666667	111,333333	4,66666667	21	18,3333333	9,33333333	0,66666667	1,66666667	30	0,33333333	0
2016	Josep Tomás	V8-V16	Late	3575	77,3333333	306,666667	3,66666667	70,3333333	6,66666667	36,6666667	1,66666667	0,66666667	169,333333	0,33333333	1,66666667
2016	Aeropuerto	V8-V16	Late	6946,33333	44,6666667	196,666667	5,33333333	56,3333333	7	109,333333	1	4,33333333	61,3333333	0,66666667	6,33333333
2016	Mirador	VT-R1	Early	322,666667	3	127,333333	8,66666667	0,66666667	4,66666667	33	2,33333333	1,33333333	0,33333333	0	0,33333333
2016	Circuito	VT-R1	Late	216	6	539,666667	6	8,66666667	0	12	1	0,66666667	63	0	1,33333333
2016	Sucs	R2-R3	Early	88	3	92	0,33333333	2,66666667	0,33333333	1,33333333	2,66666667	0,33333333	7	0,66666667	4,33333333
2016	Pantano	VT-R1	Late	204,666667	12,6666667	276	8,33333333	0,66666667	3,33333333	12,6666667	0,33333333	1,33333333	4	1,33333333	23,3333333
2016	Diarte	R2-R3	Early	90,6666667	0	92,3333333	2	2,33333333	1,33333333	2,33333333	1	0,66666667	1,33333333	0	6
2016	Aurora	VT-R1	Early	145,666667	7	719,333333	5,33333333	9,66666667	3,66666667	45,6666667	23,6666667	13	9,66666667	0	0,33333333
2016	Jardiland	V8-V16	Late	415,333333	56	301,333333	23,6666667	3	4,66666667	23,6666667	0,33333333	0	6,33333333	0	0,66666667
2016	Vencillo	VT-R1	Early	1715,33333	106,666667	155,666667	5,33333333	1,33333333	8,66666667	17,3333333	0	0,66666667	18,3333333	1	0,66666667
2016	Canal	VT-R1	Early	313,333333	18,3333333	1098,66667	45,3333333	1,66666667	9,66666667	27,3333333	0	0,33333333	0	0,33333333	0
2016	Bassanova2	R2-R3	Early	1241,66667	3,33333333	475,666667	7	0,33333333	2,33333333	6	0,33333333	0,33333333	2	0	2,33333333
2016	Ramon Vidal	R2-R3	Early	570	13,6666667	441,333333	4	0,66666667	3,33333333	22,6666667	3,33333333	7,33333333	12,6666667	0	3,33333333
2016	Gimenells	R2-R3	Early	645,333333	11	620,333333	4	29,6666667	2,66666667	35,3333333	2,33333333	11,3333333	11,6666667	0	9
2016	Miguel Velo1	VT-R1	Early	514,666667	6,33333333	967,333333	50,6666667	11,3333333	5,66666667	78,3333333	1,33333333	5,33333333	10,6666667	0	42,3333333
2016	Altorrico2	VT-R1	Late	352,333333	17,6666667	947,666667	19,6666667	2,66666667	4	38,6666667	1	1,66666667	7	1,33333333	3,66666667
2016	Canal	R4-R5	Early	162	8,66666667	5,33333333	45	19,6666667	0,66666667	45	6	0,33333333	0,66666667	0	3,33333333
2016	Altorrico1	VT-R1	Late	87	0,33333333	70	25,3333333	0,66666667	1	6	0	0,33333333	15,6666667	0	15,6666667
2016	Sucs	R4-R5	Early	11,3333333	0,33333333	16,6666667	1,33333333	0	0	2	5,33333333	0,66666667	0	0	1
2016	Altorrico2	R4-R5	Late	294	14	83,6666667	8,66666667	3	1,66666667	35,3333333	19,3333333	0,33333333	8,33333333	0,66666667	1,66666667
2016	Aeropuerto	R2-R3	Late	57	2,33333333	83	20,3333333	1	1,66666667	13,6666667	0,33333333	0	1,33333333	0,33333333	7,66666667
2016	Jardiland	R2-R3	Late	87,6666667	6	109,666667	29,3333333	3,66666667	3,66666667	22,6666667	2	2,33333333	2,33333333	0	0
2016	Ramon Vidal	R4-R5	Early	233,333333	1,33333333	177,333333	5,66666667	2,66666667	2,66666667	1,66666667	34	7,33333333	7,33333333	0	0,66666667
2016	Josep Tomás	VT-R1	Late	240,333333	13	310,333333	12	22,3333333	4,66666667	79,6666667	10,3333333	3,66666667	34,3333333	0	9,33333333

2016	Bassanova2	R6	Early	54	1,66666667	97,6666667	1,66666667	0	0,33333333	9,66666667	2,33333333	0,33333333	0	0,33333333	0
2016	Gimenells	R6	Early	175	31	105	3,33333333	74	4,33333333	28,6666667	2,33333333	13,3333333	5	0	1
2016	Alcarras-Gim1	R4-R5	Late	49	1	15	21	1,33333333	0,33333333	3,66666667	0,33333333	0	1	0	8,66666667
2016	Miguel Velo1	R2-R3	Early	60	4,66666667	33,6666667	3,66666667	1	2	17,3333333	62,6666667	1	0,66666667	0	0
2016	Manoleta	R2-R3	Early	6,33333333	0	107,666667	3,66666667	0,66666667	0,66666667	9	22	0	0,66666667	0	3,66666667
2016	Mirador	R2-R3	Early	55,3333333	1,33333333	19,6666667	1,33333333	0,66666667	2	22,6666667	11	0	0,33333333	0	0
2016	Nyaco	R2-R3	Early	192,666667	1,66666667	16,6666667	4	1	2,66666667	11	13	0	0	1,33333333	1,66666667
2016	Vencillo	R2-R3	Early	1180,33333	9	20	0,33333333	5	5,66666667	12	11,6666667	0,66666667	14,3333333	0,66666667	5,66666667
2016	Pantano	R2-R3	Late	43,3333333	8	36,6666667	6	4,33333333	5,66666667	24	42,3333333	2,66666667	2,66666667	0	11,6666667
2016	Alcarras-Gim2	R4-R5	Early	108,333333	7,66666667	30,3333333	15,3333333	2,66666667	0,66666667	7,66666667	0,66666667	0,66666667	1	0,33333333	15
2016	Alcarras	R4-R5	Late	214,666667	1	235,666667	47,6666667	4,33333333	1	4,33333333	0,66666667	2,33333333	2	0	0,66666667
2016	Miguel Velo2	R2-R3	Early	138,666667	5	13	1,66666667	4,33333333	6	5,66666667	0,66666667	0,33333333	0	0	2,66666667
2016	Diarte	R6	Early	27,3333333	0	36	1,66666667	2,66666667	0,33333333	11,3333333	2	1,33333333	0,33333333	0	1,33333333
2016	Circuito	R4-R5	Late	38,3333333	0	131	2,33333333	17,6666667	1,33333333	10,3333333	19,3333333	1,33333333	7	0	0,33333333
2016	Aurora	R4-R5	Early	38,3333333	2,66666667	199,333333	0	0,33333333	2	5,66666667	167	6	0	0	0
2016	Altorrico2	R6	Late	31,6666667	6,33333333	2,33333333	6	2	4	6,66666667	5,66666667	0	1,33333333	0,33333333	0,66666667
2016	Altorrico1	R4-R5	Late	9,33333333	1	3	5,33333333	0,33333333	1	1,66666667	11,6666667	0,33333333	0	0	1
2016	Bassanova2	R6	Early	13,6666667	0,33333333	216,333333	4,66666667	0,33333333	0	0,66666667	48,6666667	1,33333333	0,33333333	0	1,33333333
2016	Ramon Vidal	R6	Early	130	1	157	6	8,66666667	1,66666667	3,66666667	9,66666667	3	6,33333333	0	1,33333333
2016	Pantano	R6	Late	60	1,33333333	4	2,33333333	7,33333333	2	5,33333333	19,3333333	3	4,33333333	0	3,33333333
2016	Manoleta	R6	Early	7,33333333	0	4,33333333	3	1	2	14	23	4	0	0	14,6666667
2016	Alcarras-Gim1	R6	Late	8,33333333	0	2,33333333	22,3333333	1,66666667	0	2,33333333	2	0	0,33333333	0	2,33333333
2016	Alcarras-Gim2	R6	Early	24,6666667	1,33333333	8	36,3333333	7,66666667	0,66666667	5	8,33333333	0,33333333	0	0	1
2016	Aeropuerto	R2-R3	Late	1	0	20	0	4,66666667	1,33333333	4	4,33333333	0	0	0	0
2016	Circuito	R6	Late	11	0	71	0,33333333	8	1	1	12,6666667	2	0	0	0,66666667
2016	Aurora	R6	Early	8	0,66666667	120,666667	1,33333333	5	2,33333333	1	4,66666667	5,66666667	0	0	1
2016	Sucs	R4-R5	Early	5,66666667	0	22,6666667	1,33333333	0	0	0,66666667	3	0,33333333	0	0	0,33333333
2016	Miguel Velo2	R6	Early	227,666667	10,3333333	5,33333333	1,33333333	9	9	5,66666667	0,33333333	1,66666667	0	0,66666667	0,33333333

2016	Jardiland	R6	Late	59	8,66666667	14	33,3333333	5,66666667	5	7,66666667	3,66666667	3	0	0,33333333	0
2016	Vencillo	R6	Early	70,3333333	11,6666667	7,66666667	0	1,33333333	5	3,33333333	27,6666667	1	3,66666667	0,33333333	0,33333333
2016	Diarte	R4-R5	Early	12	0	13,6666667	1,33333333	0,33333333	1,33333333	1,33333333	0,66666667	5,33333333	0	0	0,33333333
2016	Mirador	R6	Early	31	5,33333333	5	2	1	2,66666667	3	33,3333333	4,33333333	0,33333333	0	0
2016	Miguel Velo1	R6	Early	12	0	3,66666667	12	0,66666667	5	6,66666667	1,33333333	0,33333333	0	0	0
2016	Josep Tomás	R2-R3	Late	16,3333333	2,66666667	13,6666667	10,3333333	3,66666667	4	30,6666667	5,66666667	1	6,33333333	0	0,33333333
2016	Alcarras	R6	Late	5	0	5,66666667	13,6666667	1,33333333	2	9	0,66666667	0,33333333	0	0	0
2016	Canal	R4-R5	Early	92	2,33333333	8,66666667	2	14,3333333	10	2,66666667	4,33333333	1,66666667	0,66666667	0,33333333	0
2016	Nyaco	R6	Early	87,3333333	4	7	7	2,33333333	3,33333333	7,66666667	3	0	0	0,33333333	2
2015	alc gim 2	V8-V16	Late	373,666667	24,6666667	0	22,3333333	0	4,66666667	0	0,66666667	0	2,33333333	0	1
2015	Josep Tomas	VT-R1	Early	525,333333	15,3333333	6,66666667	25	0	0,33333333	20,3333333	2,66666667	7	3	0	4,66666667
2015	Diarte	VT-R1	Early	554	27	14,3333333	22,6666667	0	0,33333333	2,33333333	12,6666667	0,33333333	3	0	5
2015	Nyaco	V8-V16	Late	2585	57	113,333333	16	0	10,6666667	4	0,66666667	2,33333333	11	0	0,66666667
2015	Bassanova 2	V8-V16	Early	119	10,3333333	53,6666667	28,3333333	0	0,33333333	2,66666667	1	0	1,33333333	0	4
2015	Vencillo	V8-V16	Late	1982,66667	63,3333333	5,66666667	7,33333333	0	2,33333333	6	0,66666667	0,66666667	5,66666667	0,33333333	3,33333333
2015	alc gim 2	VT-R1	Late	222,666667	6,33333333	207	48,3333333	0	1,66666667	2,33333333	7	0,33333333	0,33333333	0	8,33333333
2015	Josep Tomas	R2-R3	Early	627,333333	4,33333333	306,333333	9	0	2,33333333	16,3333333	0,33333333	7	19	0,33333333	3,66666667
2015	Diarte	R2-R3	Early	312,666667	2	93,3333333	3,66666667	0	2,33333333	2,33333333	6,33333333	0	0,66666667	0,33333333	2,33333333
2015	Nyaco	V8-V16	Late	629,666667	6	51	25,6666667	0	0,66666667	4	1,66666667	1,33333333	2,33333333	0,33333333	1,33333333
2015	Bassanova 2	R2-R3	Early	171,666667	2	1368,33333	8	0	0,33333333	2	1	4,33333333	1	0	2
2015	Vencillo	VT-R1	Late	72	0,33333333	53	8,33333333	0	0	1,33333333	1,33333333	0	0	0	0,66666667
2015	alc gim 2	R4-R5	Late	281,666667	5	64,3333333	54	0	1,33333333	0	0	1	0	0,33333333	0
2015	Josep Tomas	R4-R5	Early	288	4,33333333	164	2,33333333	0	1	14	1	2,66666667	4,33333333	0,66666667	5,66666667
2015	Diarte	R4-R5	Early	23,6666667	3,33333333	80	3,33333333	0	1,66666667	1,33333333	0	5	0,33333333	0	2,33333333
2015	Nyaco	R2-R3	Late	29,6666667	0,33333333	21,6666667	7,33333333	0	0,33333333	1,66666667	11	0,33333333	0	0	0
2015	Bassanova 2	R4-R5	Early	150	1,33333333	116,333333	5	0	0,33333333	1,66666667	1	4	1,33333333	0	1,66666667
2015	Vencillo	R4-R5	Late	42,6666667	2,33333333	6,33333333	1,66666667	0	0,66666667	2,66666667	0,33333333	0,33333333	0	0	1
2015	alc gim 2	R6	Late	19,6666667	4	3,66666667	18,3333333	0	2,66666667	0,33333333	0,33333333	4	0,66666667	1	0,33333333

2015	Josep Tomas	R6	Early	60,6666667	8	14	2,33333333	0	17,3333333	3	0,33333333	1,33333333	0	0,33333333	4,66666667
2015	Diarte	R6	Early	21,6666667	0,33333333	8	4,33333333	0	3,33333333	0,33333333	0	5,33333333	0	0	0,33333333
2015	Nyaco	R6	Late	25,6666667	0	4,66666667	5,66666667	0	0,33333333	0,33333333	0,33333333	0	0	0	0,33333333
2015	Bassanova 2	R6	Early	10,6666667	1	20	10	0	0,33333333	1	0,66666667	4,33333333	0	0	0
2015	Vencillo	R6	Late	15	1,33333333	2,66666667	0,66666667	0	1	0,33333333	0	0,33333333	0,33333333	0	0,33333333

S2. Extra model results regarding Figure 2. Poshocs results.

Orius sp

Estimate Std. Error z value Pr(>|z|)
(R2-R3) - (R4-R5) == 0 0.1129 0.2328 0.485 0.9967
(R2-R3) - R6 == 0 0.6864 0.2285 3.004 0.0317 *
(R2-R3) - (V1-V7) == 0 1.2989 0.2649 4.904 <0.001 ***
(R2-R3) - (V8-V16) == 0 -0.3218 0.2309 -1.394 0.7296
(R2-R3) - (VT-R1) == 0 -0.6264 0.2282 -2.745 0.0662 .
(R4-R5) - R6 == 0 0.5735 0.2257 2.541 0.1115
(R4-R5) - (V1-V7) == 0 1.1860 0.2554 4.644 <0.001 ***
(R4-R5) - (V8-V16) == 0 -0.4347 0.2224 -1.954 0.3672
(R4-R5) - (VT-R1) == 0 -0.7393 0.2224 -3.325 0.0113 *
R6 - (V1-V7) == 0 0.6125 0.2624 2.334 0.1791
R6 - (V8-V16) == 0 -1.0082 0.2266 -4.450 <0.001 ***
R6 - (VT-R1) == 0 -1.3127 0.2264 -5.798 <0.001 ***
(V1-V7) - (V8-V16) == 0 -1.6207 0.2665 -6.081 <0.001 ***
(V1-V7) - (VT-R1) == 0 -1.9252 0.2547 -7.558 <0.001 ***
(V8-V16) - (VT-R1) == 0 -0.3046 0.2305 -1.322
0.7719

Syrfidae

Estimate Std. Error z value Pr(>|z|)
(R2-R3) - (R4-R5) == 0 -0.24322 0.83070 -0.293 0.99968
(R2-R3) - R6 == 0 -0.31737 0.83962 -0.378 0.99889
(R2-R3) - (V1-V7) == 0 -3.08398 0.71442 -4.317 < 0.001 ***
(R2-R3) - (V8-V16) == 0 -1.58399 0.77350 -2.048 0.29797
(R2-R3) - (VT-R1) == 0 -0.43897 0.82782 -0.530 0.99441
(R4-R5) - R6 == 0 -0.07415 0.66785 -0.111 1.00000
(R4-R5) - (V1-V7) == 0 -2.84076 0.49707 -5.715 < 0.001 ***
(R4-R5) - (V8-V16) == 0 -1.34077 0.59090 -2.269 0.19362
(R4-R5) - (VT-R1) == 0 -0.19575 0.65441 -0.299 0.99964

R6 - (V1-V7) == 0 -2.76661 0.52078 -5.312 < 0.001 ***
R6 - (V8-V16) == 0 -1.26662 0.59571 -2.126 0.25805
R6 - (VT-R1) == 0 -0.12160 0.66490 -0.183 0.99997
(V1-V7) - (V8-V16) == 0 1.49999 0.41730 3.595 0.00424 **
(V1-V7) - (VT-R1) == 0 2.64501 0.50061 5.284 < 0.001 ***
(V8-V16) - (VT-R1) == 0 1.14502 0.58939 1.943
0.35752

Aeolothrips sp
Estimate Std. Error z value Pr(>|z|)
(R2-R3) - (R4-R5) == 0 0.8315 0.3403 2.444 0.140
(R2-R3) - R6 == 0 1.5343 0.3741 4.102 <0.001 ***
(R2-R3) - (V1-V7) == 0 -2.1936 0.3461 -6.339 <0.001 ***
(R2-R3) - (V8-V16) == 0 -1.9207 0.3245 -5.919 <0.001 ***
(R2-R3) - (VT-R1) == 0 -1.5369 0.3319 -4.631 <0.001 ***
(R4-R5) - R6 == 0 0.7028 0.3769 1.865 0.422
(R4-R5) - (V1-V7) == 0 -3.0251 0.3346 -9.041 <0.001 ***
(R4-R5) - (V8-V16) == 0 -2.7522 0.3251 -8.466 <0.001 ***
(R4-R5) - (VT-R1) == 0 -2.3684 0.3268 -7.247 <0.001 ***
R6 - (V1-V7) == 0 -3.7280 0.3789 -9.840 <0.001 ***
R6 - (V8-V16) == 0 -3.4550 0.3540 -9.761 <0.001 ***
R6 - (VT-R1) == 0 -3.0712 0.3634 -8.450 <0.001 ***
(V1-V7) - (V8-V16) == 0 0.2730 0.3332 0.819 0.964
(V1-V7) - (VT-R1) == 0 0.6567 0.3155 2.082 0.295
(V8-V16) - (VT-R1) == 0 0.3838 0.3101 1.237
0.817

Coccinellidae
Estimate Std. Error z value Pr(>|z|)
(R2-R3) - (R4-R5) == 0 0.7145 0.3135 2.279 0.2014

(R2-R3) - R6 == 0 1.3100 0.3253 4.027 <0.001 ***
(R2-R3) - (V1-V7) == 0 0.3692 0.3290 1.122 0.8717
(R2-R3) - (V8-V16) == 0 0.3968 0.3052 1.300 0.7842
(R2-R3) - (VT-R1) == 0 -0.2792 0.2905 -0.961 0.9297
(R4-R5) - R6 == 0 0.5955 0.3406 1.748 0.4981
(R4-R5) - (V1-V7) == 0 -0.3454 0.3419 -1.010 0.9143
(R4-R5) - (V8-V16) == 0 -0.3178 0.3181 -0.999 0.9179
(R4-R5) - (VT-R1) == 0 -0.9937 0.3113 -3.193 0.0177 *
R6 - (V1-V7) == 0 -0.9408 0.3669 -2.564 0.1057
R6 - (V8-V16) == 0 -0.9132 0.3340 -2.734 0.0682 .
R6 - (VT-R1) == 0 -1.5892 0.3222 -4.932 <0.001 ***
(V1-V7) - (V8-V16) == 0 0.0276 0.3437 0.080 1.0000
(V1-V7) - (VT-R1) == 0 -0.6484 0.3313 -1.957 0.3656
(V8-V16) - (VT-R1) == 0 -0.6760 0.3009 -2.247
0.2152

Sthethorus
Estimate Std. Error z value Pr(>|z|)
(R2-R3) - (R4-R5) == 0 -0.51703 0.34535 -1.497 0.6619
(R2-R3) - R6 == 0 -0.01492 0.32720 -0.046 1.0000
(R2-R3) - (V1-V7) == 0 2.25775 0.39468 5.720 <0.001 ***
(R2-R3) - (V8-V16) == 0 3.03252 0.42431 7.147 <0.001 ***
(R2-R3) - (VT-R1) == 0 1.10045 0.36595 3.007 0.0310 *
(R4-R5) - R6 == 0 0.50211 0.32030 1.568 0.6157
(R4-R5) - (V1-V7) == 0 2.77478 0.37549 7.390 <0.001 ***
(R4-R5) - (V8-V16) == 0 3.54955 0.40690 8.723 <0.001 ***
(R4-R5) - (VT-R1) == 0 1.61748 0.33798 4.786 <0.001 ***
R6 - (V1-V7) == 0 2.27267 0.37568 6.049 <0.001 ***
R6 - (V8-V16) == 0 3.04744 0.40936 7.444 <0.001 ***
R6 - (VT-R1) == 0 1.11537 0.34777 3.207 0.0166 *

(V1-V7) - (V8-V16) == 0 0.77477   0.46357   1.671   0.5461
(V1-V7) - (VT-R1) == 0 -1.15730   0.40533 -2.855   0.0482 *
(V8-V16) - (VT-R1) == 0 -1.93207   0.43245 -4.468
<0.001 ***

F. occidentalis
Estimate Std. Error z value Pr(>|z|)
(R2-R3) - (R4-R5) == 0   0.5456   0.2589   2.107 0.28287
(R2-R3) - R6 == 0   1.3207   0.2628   5.025 < 0.001 ***
(R2-R3) - (V1-V7) == 0   -0.5881   0.2820 -2.086 0.29391
(R2-R3) - (V8-V16) == 0 -1.5379   0.2773 -5.545 < 0.001 ***
(R2-R3) - (VT-R1) == 0   -0.4749   0.2696 -1.762 0.48983
(R4-R5) - R6 == 0   0.7751   0.2519   3.077 0.02551 *
(R4-R5) - (V1-V7) == 0   -1.1337   0.2706 -4.190 < 0.001 ***
(R4-R5) - (V8-V16) == 0 -2.0835   0.2647 -7.872 < 0.001 ***
(R4-R5) - (VT-R1) == 0   -1.0205   0.2533 -4.029 < 0.001 ***
R6 - (V1-V7) == 0   -1.9089   0.2734 -6.982 < 0.001 ***
R6 - (V8-V16) == 0   -2.8586   0.2693 -10.615 < 0.001 ***
R6 - (VT-R1) == 0   -1.7957   0.2564 -7.005 < 0.001 ***
(V1-V7) - (V8-V16) == 0 -0.9498   0.2941 -3.229 0.01566 *
(V1-V7) - (VT-R1) == 0   0.1132   0.2786   0.406 0.99858
(V8-V16) - (VT-R1) == 0   1.0630   0.2713   3.918
0.00126 **

Other herbivore thrips
Estimate Std. Error z value Pr(>|z|)
(R2-R3) - (R4-R5) == 0   0.1860   0.2577   0.722   0.9793
(R2-R3) - R6 == 0   0.3799   0.2565   1.481   0.6758
(R2-R3) - (V1-V7) == 0   -3.6892   0.2499 -14.763   <0.001 ***
(R2-R3) - (V8-V16) == 0 -2.9386   0.2488 -11.811   <0.001 ***
(R2-R3) - (VT-R1) == 0   -1.0333   0.2507 -4.121   <0.001 ***

(R4-R5) - R6 == 0 0.1939 0.2522 0.769 0.9726
(R4-R5) - (V1-V7) == 0 -3.8752 0.2445 -15.851 <0.001 ***
(R4-R5) - (V8-V16) == 0 -3.1246 0.2369 -13.191 <0.001 ***
(R4-R5) - (VT-R1) == 0 -1.2193 0.2449 -4.979 <0.001 ***
R6 - (V1-V7) == 0 -4.0692 0.2507 -16.234 <0.001 ***
R6 - (V8-V16) == 0 -3.3185 0.2429 -13.662 <0.001 ***
R6 - (VT-R1) == 0 -1.4132 0.2400 -5.888 <0.001 ***
(V1-V7) - (V8-V16) == 0 0.7506 0.2247 3.341 0.0107 *
(V1-V7) - (VT-R1) == 0 2.6560 0.2406 11.038 <0.001 ***
(V8-V16) - (VT-R1) == 0 1.9053 0.2353 8.099
<0.001 ***

Z. scutellaris

Estimate Std. Error z value Pr(>|z|)
(R2-R3) - (R4-R5) == 0 0.996924 0.278337 3.582 0.00458 **
(R2-R3) - R6 == 0 1.998278 0.286943 6.964 < 0.001 ***
(R2-R3) - (V1-V7) == 0 0.002275 0.322001 0.007 1.00000
(R2-R3) - (V8-V16) == 0 -0.169626 0.298671 -0.568 0.99302
(R2-R3) - (VT-R1) == 0 -1.309646 0.300382 -4.360 < 0.001 ***
(R4-R5) - R6 == 0 1.001354 0.268483 3.730 0.00265 **
(R4-R5) - (V1-V7) == 0 -0.994649 0.303496 -3.277 0.01335 *
(R4-R5) - (V8-V16) == 0 -1.166551 0.278616 -4.187 < 0.001 ***
(R4-R5) - (VT-R1) == 0 -2.306570 0.284136 -8.118 < 0.001 ***
R6 - (V1-V7) == 0 -1.996002 0.315190 -6.333 < 0.001 ***
R6 - (V8-V16) == 0 -2.167904 0.286259 -7.573 < 0.001 ***
R6 - (VT-R1) == 0 -3.307923 0.290076 -11.404 < 0.001 ***
(V1-V7) - (V8-V16) == 0 -0.171902 0.315419 -0.545 0.99424
(V1-V7) - (VT-R1) == 0 -1.311921 0.302628 -4.335 < 0.001 ***
(V8-V16) - (VT-R1) == 0 -1.140019 0.292691 -3.895
0.00146 **

Aphid

Estimate Std. Error z value Pr(>|z|)
(R2-R3) - (R4-R5) == 0   0.34619   0.27536   1.257   0.8062
(R2-R3) - R6 == 0   -0.32471   0.24023 -1.352   0.7535
(R2-R3) - (V1-V7) == 0 -3.97175   0.24176 -16.429   <0.001 ***
(R2-R3) - (V8-V16) == 0 -1.74977   0.22615 -7.737   <0.001 ***
(R2-R3) - (VT-R1) == 0 -0.39676   0.24853 -1.596   0.5979
(R4-R5) - R6 == 0   -0.67090   0.25370 -2.644   0.0858 .
(R4-R5) - (V1-V7) == 0 -4.31794   0.24122 -17.900   <0.001 ***
(R4-R5) - (V8-V16) == 0 -2.09596   0.24603 -8.519   <0.001 ***
(R4-R5) - (VT-R1) == 0 -0.74295   0.26059 -2.851   0.0490 *
R6 - (V1-V7) == 0   -3.64704   0.21494 -16.968   <0.001 ***
R6 - (V8-V16) == 0   -1.42506   0.20578 -6.925   <0.001 ***
R6 - (VT-R1) == 0   -0.07205   0.22490 -0.320   0.9995
(V1-V7) - (V8-V16) == 0 2.22198   0.21443 10.362   <0.001 ***
(V1-V7) - (VT-R1) == 0   3.57499   0.22166 16.128   <0.001 ***
(V8-V16) - (VT-R1) == 0 1.35301   0.21653   6.249
<0.001 ***

Empoasca sp.

Estimate Std. Error z value Pr(>|z|)
(R2-R3) - (R4-R5) == 0   0.07574   0.30375   0.249 0.99987
(R2-R3) - R6 == 0   -0.05898   0.30783 -0.192 0.99996
(R2-R3) - (V1-V7) == 0 -0.48186   0.31477 -1.531 0.64368
(R2-R3) - (V8-V16) == 0 -0.97432   0.31702 -3.073 0.02583 *
(R2-R3) - (VT-R1) == 0 -0.20018   0.31075 -0.644 0.98759
(R4-R5) - R6 == 0   -0.13472   0.29603 -0.455 0.99755
(R4-R5) - (V1-V7) == 0 -0.55760   0.29960 -1.861 0.42587
(R4-R5) - (V8-V16) == 0 -1.05006   0.30433 -3.450 0.00743 **
(R4-R5) - (VT-R1) == 0 -0.27592   0.29653 -0.930 0.93860
R6 - (V1-V7) == 0   -0.42288   0.30812 -1.372 0.74322

R6 - (V8-V16) == 0 -0.91534 0.31004 -2.952 0.03724 *
R6 - (VT-R1) == 0 -0.14120 0.30786 -0.459 0.99746
(V1-V7) - (V8-V16) == 0 -0.49245 0.33109 -1.487 0.67197
(V1-V7) - (VT-R1) == 0 0.28168 0.30993 0.909 0.94425
(V8-V16) - (VT-R1) == 0 0.77414 0.31752 2.438
0.14311

L. striatellus
Linear Hypotheses:
Estimate Std. Error z value Pr(>|z|)
(R2-R3) - (R4-R5) == 0 0.03101 0.25595 0.121 1.00000
(R2-R3) - R6 == 0 0.49043 0.25581 1.917 0.39040
(R2-R3) - (V1-V7) == 0 0.20299 0.27984 0.725 0.97879
(R2-R3) - (V8-V16) == 0 -0.98752 0.26141 -3.778 0.00226 **
(R2-R3) - (VT-R1) == 0 -1.01058 0.26059 -3.878 0.00147 **
(R4-R5) - R6 == 0 0.45941 0.23753 1.934 0.38005
(R4-R5) - (V1-V7) == 0 0.17198 0.26044 0.660 0.98608
(R4-R5) - (V8-V16) == 0 -1.01854 0.24616 -4.138 < 0.001 ***
(R4-R5) - (VT-R1) == 0 -1.04159 0.24530 -4.246 < 0.001 ***
R6 - (V1-V7) == 0 -0.28744 0.27186 -1.057 0.89775
R6 - (V8-V16) == 0 -1.47795 0.25063 -5.897 < 0.001 ***
R6 - (VT-R1) == 0 -1.50101 0.25111 -5.977 < 0.001 ***
(V1-V7) - (V8-V16) == 0 -1.19051 0.27906 -4.266 < 0.001 ***
(V1-V7) - (VT-R1) == 0 -1.21357 0.27199 -4.462 < 0.001 ***
(V8-V16) - (VT-R1) == 0 -0.02306 0.25238 -0.091
1.00000

S3. Extra model results regarding Figure 3 and 4. Poshoc.

Early date_models, were letters means: a= 2015, b= 2016, c= 2017

Response: **Laodelphax**

```
      Chisq Df Pr(>Chisq)
year 9.6093  2   0.008192 **
---
Signif. codes:  0 ‘***’ 0.001 ‘**’ 0.01 ‘*’ 0.05 ‘.’ 0.1 ‘ ’ 1
> mod.emm <- emmeans(m3, ~ year)
> mod.emm
 year emmean    SE  df asymp.LCL asymp.UCL
 a      2.35 0.380 Inf      1.61       3.1
 b      2.33 0.291 Inf      1.76       2.9
 c      1.83 0.292 Inf      1.25       2.4

Results are given on the log (not the response) scale.
Confidence level used: 0.95
> summary(as.glht(pairs(mod.emm)))

         Simultaneous Tests for General Linear Hypotheses

Linear Hypotheses:
           Estimate Std. Error z value Pr(>|z|)
a - b == 0  0.01875    0.30639   0.061  0.99786
a - c == 0  0.52559    0.31516   1.668  0.20830
b - c == 0  0.50684    0.16849   3.008  0.00687 **
```

Response: **F. occidentalis**

```
      Chisq Df Pr(>Chisq)
year 41.374  2  1.037e-09 ***
```

```
---
Signif. codes:  0 ‘***’ 0.001 ‘**’ 0.01 ‘*’ 0.05 ‘.’ 0.1 ‘ ’ 1
> mod.emm <- emmeans(m3, ~ year)
> mod.emm
 year emmean    SE  df asymp.LCL asymp.UCL
 a      5.40 0.446 Inf      4.52      6.27
 b      5.59 0.392 Inf      4.82      6.36
 c      4.45 0.392 Inf      3.68      5.22

Results are given on the log (not the response) scale.
Confidence level used: 0.95
> summary(as.glht(pairs(mod.emm)))

	 Simultaneous Tests for General Linear Hypotheses

Linear Hypotheses:
           Estimate Std. Error z value Pr(>|z|)
a - b == 0  -0.1949     0.2752  -0.708  0.75403
a - c == 0   0.9461     0.2816   3.360  0.00213 **
b - c == 0   1.1410     0.1805   6.321  < 1e-05 ***
---
Signif. codes:  0 ‘***’ 0.001 ‘**’ 0.01 ‘*’ 0.05 ‘.’ 0.1 ‘ ’ 1
(Adjusted p values reported -- single-step method)

> multcomp::cld(mod.emm, alpha = 0.05, Letters = LETTERS) ##agrega las letras al poshoc
 year emmean    SE  df asymp.LCL asymp.UCL .group
 c      4.45 0.392 Inf      3.68      5.22  A    
 a      5.40 0.446 Inf      4.52      6.27   B   
 b      5.59 0.392 Inf      4.82      6.36   B   

Results are given on the log (not the response) scale.
```

```
Confidence level used: 0.95
P value adjustment: tukey method for comparing a family of 3 estimates
significance level used: alpha = 0.05
```

Response: **Other herbivore thrips**

```
     Chisq Df Pr(>Chisq)
year 4.2073  2      0.122
> mod.emm <- emmeans(m3, ~ year)
> mod.emm
 year emmean    SE  df asymp.LCL asymp.UCL
 a      2.06 0.678 Inf     0.728      3.39
 b      2.65 0.636 Inf     1.405      3.90
 c      2.63 0.635 Inf     1.385      3.87

Results are given on the log (not the response) scale.
Confidence level used: 0.95
> summary(as.glht(pairs(mod.emm)))

         Simultaneous Tests for General Linear Hypotheses

Linear Hypotheses:
           Estimate Std. Error z value Pr(>|z|)
a - b == 0  -0.5941     0.2945  -2.017    0.103
a - c == 0  -0.5717     0.3024  -1.890    0.135
b - c == 0   0.0224     0.1713   0.131    0.990
(Adjusted p values reported -- single-step method)

> multcomp::cld(mod.emm, alpha = 0.05, Letters = LETTERS) ##agrega las letras al poshoc
 year emmean    SE  df asymp.LCL asymp.UCL .group
 a      2.06 0.678 Inf     0.728      3.39  A
```

```
 c      2.63 0.635 Inf     1.385      3.87  A
 b      2.65 0.636 Inf     1.405      3.90  A

Results are given on the log (not the response) scale.
Confidence level used: 0.95
P value adjustment: tukey method for comparing a family of 3 estimates
significance level used: alpha = 0.05
```

Response: **Z. scutellaris**

```
      Chisq Df Pr(>Chisq)
year 39.217  2  3.049e-09 ***
---
Signif. codes:  0 ‘***’ 0.001 ‘**’ 0.01 ‘*’ 0.05 ‘.’ 0.1 ‘ ’ 1
> mod.emm <- emmeans(m3, ~ year)
> mod.emm
 year emmean    SE  df asymp.LCL asymp.UCL
 a      4.46 0.533 Inf      3.42      5.51
 b      4.78 0.458 Inf      3.88      5.68
 c      3.63 0.457 Inf      2.73      4.52

Results are given on the log (not the response) scale.
Confidence level used: 0.95
> summary(as.glht(pairs(mod.emm)))

         Simultaneous Tests for General Linear Hypotheses

Linear Hypotheses:
           Estimate Std. Error z value Pr(>|z|)
a - b == 0  -0.3174     0.3468  -0.915   0.6205
a - c == 0   0.8354     0.3474   2.405   0.0399 *
```

```
b - c == 0   1.1527     0.1854   6.217   <1e-04 ***
---
Signif. codes:  0 ‘***’ 0.001 ‘**’ 0.01 ‘*’ 0.05 ‘.’ 0.1 ‘ ’ 1
(Adjusted p values reported -- single-step method)

> multcomp::cld(mod.emm, alpha = 0.05, Letters = LETTERS) ##agrega las letras al poshoc
 year emmean    SE  df asymp.LCL asymp.UCL .group
 c      3.63 0.457 Inf      2.73      4.52  A
 a      4.46 0.533 Inf      3.42      5.51   B
 b      4.78 0.458 Inf      3.88      5.68   B

Results are given on the log (not the response) scale.
Confidence level used: 0.95
P value adjustment: tukey method for comparing a family of 3 estimates
significance level used: alpha = 0.05
```

Response: **Empoasca sp.**

```
       Chisq Df Pr(>Chisq)
year 10265748  2  < 2.2e-16 ***
---
Signif. codes:  0 ‘***’ 0.001 ‘**’ 0.01 ‘*’ 0.05 ‘.’ 0.1 ‘ ’ 1
> mod.emm <- emmeans(m3, ~ year)
> mod.emm
 year  emmean       SE  df asymp.LCL asymp.UCL
 a    -16.987 0.008396 Inf   -17.003   -16.970
 b      1.827 0.011868 Inf     1.804     1.850
 c      2.259 0.011867 Inf     2.236     2.282

Results are given on the log (not the response) scale.
Confidence level used: 0.95
```

```
> summary(as.glht(pairs(mod.emm)))

	 Simultaneous Tests for General Linear Hypotheses

Linear Hypotheses:
             Estimate Std. Error  z value Pr(>|z|)
a - b == 0 -18.813594   0.008394 -2241.22   <2e-16 ***
a - c == 0 -19.245705   0.008394 -2292.82   <2e-16 ***
b - c == 0  -0.432111   0.011863   -36.43   <2e-16 ***
---
Signif. codes:  0 ‘***’ 0.001 ‘**’ 0.01 ‘*’ 0.05 ‘.’ 0.1 ‘ ’ 1
(Adjusted p values reported -- single-step method)

> multcomp::cld(mod.emm, alpha = 0.05, Letters = LETTERS) ##agrega las letras al poshoc
 year  emmean       SE  df asymp.LCL asymp.UCL .group
 a    -16.987 0.008396 Inf   -17.003   -16.970  A
 b      1.827 0.011868 Inf     1.804     1.850   B
 c      2.259 0.011867 Inf     2.236     2.282    C

Results are given on the log (not the response) scale.
Confidence level used: 0.95
P value adjustment: tukey method for comparing a family of 3 estimates
significance level used: alpha = 0.05
```

Response: **Aphid**

```
      Chisq Df Pr(>Chisq)
year 15.437  2  0.0004445 ***
---
Signif. codes:  0 ‘***’ 0.001 ‘**’ 0.01 ‘*’ 0.05 ‘.’ 0.1 ‘ ’ 1
> mod.emm <- emmeans(m3, ~ year)
> mod.emm
```

```
 year emmean    SE  df asymp.LCL asymp.UCL
 a       1.04 0.649 Inf    -0.228      2.31
 b       1.96 0.604 Inf     0.772      3.14
 c       1.53 0.605 Inf     0.347      2.72
```

Response: **Orius sp.**

```
      Chisq Df Pr(>Chisq)
year 40.548  2  1.567e-09 ***
---
Signif. codes:  0 ‘***’ 0.001 ‘**’ 0.01 ‘*’ 0.05 ‘.’ 0.1 ‘ ’ 1
> mod.emm <- emmeans(m3, ~ year)
> mod.emm
 year emmean    SE  df asymp.LCL asymp.UCL
 a     0.809 0.373 Inf    0.0775      1.54
 b     2.527 0.285 Inf    1.9681      3.09
 c     2.037 0.286 Inf    1.4768      2.60

Results are given on the log (not the response) scale.
Confidence level used: 0.95
> summary(as.glht(pairs(mod.emm)))

         Simultaneous Tests for General Linear Hypotheses

Linear Hypotheses:
           Estimate Std. Error z value Pr(>|z|)
a - b == 0  -1.7179     0.2849  -6.029  < 1e-04 ***
a - c == 0  -1.2273     0.2891  -4.245  < 1e-04 ***
b - c == 0   0.4905     0.1458   3.363  0.00204 **
---
```

```
Signif. codes:  0 ‘***’ 0.001 ‘**’ 0.01 ‘*’ 0.05 ‘.’ 0.1 ‘ ’ 1
(Adjusted p values reported -- single-step method)

> multcomp::cld(mod.emm, alpha = 0.05, Letters = LETTERS) ##agrega las letras al poshoc
 year emmean    SE  df asymp.LCL asymp.UCL .group
 a     0.809 0.373 Inf    0.0775      1.54  A
 c     2.037 0.286 Inf    1.4768      2.60   B
 b     2.527 0.285 Inf    1.9681      3.09    C

Results are given on the log (not the response) scale.
Confidence level used: 0.95
P value adjustment: tukey method for comparing a family of 3 estimates
significance level used: alpha = 0.05
```

Response: **Aeolothrips sp.**

```
      Chisq Df Pr(>Chisq)
year 11.705  2   0.002873 **
---
Signif. codes:  0 ‘***’ 0.001 ‘**’ 0.01 ‘*’ 0.05 ‘.’ 0.1 ‘ ’ 1
> mod.emm <- emmeans(m3, ~ year)
> mod.emm
 year emmean    SE  df asymp.LCL asymp.UCL
 a     0.584 0.708 Inf    -0.804      1.97
 b     1.861 0.612 Inf     0.662      3.06
 c     1.375 0.611 Inf     0.177      2.57

Results are given on the log (not the response) scale.
Confidence level used: 0.95
> summary(as.glht(pairs(mod.emm)))
```

```
	 Simultaneous Tests for General Linear Hypotheses

Linear Hypotheses:
           Estimate Std. Error z value Pr(>|z|)
a - b == 0  -1.2769     0.4234  -3.016   0.0066 **
a - c == 0  -0.7905     0.4325  -1.828   0.1517
b - c == 0   0.4864     0.2184   2.227   0.0621 .
---
Signif. codes:  0 ‘***’ 0.001 ‘**’ 0.01 ‘*’ 0.05 ‘.’ 0.1 ‘ ’ 1
(Adjusted p values reported -- single-step method)

> multcomp::cld(mod.emm, alpha = 0.05, Letters = LETTERS) ##agrega las letras al poshoc
 year emmean    SE  df asymp.LCL asymp.UCL .group
 a     0.584 0.708 Inf    -0.804      1.97  A
 c     1.375 0.611 Inf     0.177      2.57  AB
 b     1.861 0.612 Inf     0.662      3.06   B

Results are given on the log (not the response) scale.
Confidence level used: 0.95
P value adjustment: tukey method for comparing a family of 3 estimates
significance level used: alpha = 0.05
```

Response: **Coccinellidae**

```
      Chisq Df Pr(>Chisq)
year 19.512  2  5.794e-05 ***
---
Signif. codes:  0 ‘***’ 0.001 ‘**’ 0.01 ‘*’ 0.05 ‘.’ 0.1 ‘ ’ 1
> mod.emm <- emmeans(m3, ~ year)
> mod.emm
 year emmean    SE  df asymp.LCL asymp.UCL
```

```
 a     0.706 0.354 Inf    0.0118     1.401
 b     1.034 0.234 Inf    0.5748     1.494
 c     0.160 0.243 Inf   -0.3172     0.637

Results are given on the log (not the response) scale.
Confidence level used: 0.95
> summary(as.glht(pairs(mod.emm)))

         Simultaneous Tests for General Linear Hypotheses

Linear Hypotheses:
           Estimate Std. Error z value Pr(>|z|)
a - b == 0  -0.3277     0.3348  -0.979    0.581
a - c == 0   0.5465     0.3483   1.569    0.250
b - c == 0   0.8742     0.1979   4.417   <1e-04 ***
---
Signif. codes:  0 ‘***’ 0.001 ‘**’ 0.01 ‘*’ 0.05 ‘.’ 0.1 ‘ ’ 1
(Adjusted p values reported -- single-step method)

> multcomp::cld(mod.emm, alpha = 0.05, Letters = LETTERS) ##agrega las letras al poshoc
 year emmean    SE  df asymp.LCL asymp.UCL .group
 c     0.160 0.243 Inf   -0.3172     0.637  A
 a     0.706 0.354 Inf    0.0118     1.401  AB
 b     1.034 0.234 Inf    0.5748     1.494   B

Results are given on the log (not the response) scale.
Confidence level used: 0.95
P value adjustment: tukey method for comparing a family of 3 estimates
significance level used: alpha = 0.05
```

Late date_models were letters means: a= 2015, b= 2016, c= 2017

```
Response: F. occidentalis
      Chisq Df Pr(>Chisq)
year 41.374  2  1.037e-09 ***
---
Signif. codes:  0 ‘***’ 0.001 ‘**’ 0.01 ‘*’ 0.05 ‘.’ 0.1 ‘ ’ 1
> mod.emm <- emmeans(m3, ~ year)
> mod.emm
 year emmean    SE  df asymp.LCL asymp.UCL
 a      5.40 0.446 Inf      4.52      6.27
 b      5.59 0.392 Inf      4.82      6.36
 c      4.45 0.392 Inf      3.68      5.22

Results are given on the log (not the response) scale.
Confidence level used: 0.95
> summary(as.glht(pairs(mod.emm)))

         Simultaneous Tests for General Linear Hypotheses

Linear Hypotheses:
           Estimate Std. Error z value Pr(>|z|)
a - b == 0  -0.1949     0.2752  -0.708  0.75402
a - c == 0   0.9461     0.2816   3.360  0.00215 **
b - c == 0   1.1410     0.1805   6.321  < 1e-04 ***
---
Signif. codes:  0 ‘***’ 0.001 ‘**’ 0.01 ‘*’ 0.05 ‘.’ 0.1 ‘ ’ 1
(Adjusted p values reported -- single-step method)
```

```
> multcomp::cld(mod.emm, alpha = 0.05, Letters = LETTERS) ##agrega las letras al poshoc
 year emmean    SE  df asymp.LCL asymp.UCL .group
 c      4.45 0.392 Inf      3.68      5.22  A
 a      5.40 0.446 Inf      4.52      6.27   B
 b      5.59 0.392 Inf      4.82      6.36   B

Results are given on the log (not the response) scale.
Confidence level used: 0.95
P value adjustment: tukey method for comparing a family of 3 estimates
significance level used: alpha = 0.05
```

Response: **other herbivore thrips**

```
      Chisq Df Pr(>Chisq)
year 4.2073  2      0.122
```

Response: **Z. scutellaris**

```
      Chisq Df Pr(>Chisq)
year 39.217  2  3.049e-09 ***
---
Signif. codes:  0 '***' 0.001 '**' 0.01 '*' 0.05 '.' 0.1 ' ' 1
> mod.emm <- emmeans(m3, ~ year)
> mod.emm
 year emmean    SE  df asymp.LCL asymp.UCL
 a      4.46 0.533 Inf      3.42      5.51
 b      4.78 0.458 Inf      3.88      5.68
 c      3.63 0.457 Inf      2.73      4.52
```

```
Results are given on the log (not the response) scale.
Confidence level used: 0.95
> summary(as.glht(pairs(mod.emm)))

	 Simultaneous Tests for General Linear Hypotheses

Linear Hypotheses:
           Estimate Std. Error z value Pr(>|z|)
a - b == 0  -0.3174     0.3468  -0.915     0.62
a - c == 0   0.8354     0.3474   2.405     0.04 *
b - c == 0   1.1527     0.1854   6.217   <1e-04 ***
---
Signif. codes:  0 ‘***’ 0.001 ‘**’ 0.01 ‘*’ 0.05 ‘.’ 0.1 ‘ ’ 1
(Adjusted p values reported -- single-step method)

> multcomp::cld(mod.emm, alpha = 0.05, Letters = LETTERS) ##agrega las letras al
poshoc
 year emmean    SE  df asymp.LCL asymp.UCL .group
 c      3.63 0.457 Inf      2.73      4.52  A
 a      4.46 0.533 Inf      3.42      5.51   B
 b      4.78 0.458 Inf      3.88      5.68   B

Results are given on the log (not the response) scale.
Confidence level used: 0.95
P value adjustment: tukey method for comparing a family of 3 estimates
significance level used: alpha = 0.05
```

Response: **Empoasca sp.**

```
        Chisq Df Pr(>Chisq)
year 10265748  2  < 2.2e-16 ***
```

```
---
Signif. codes:  0 ‘***’ 0.001 ‘**’ 0.01 ‘*’ 0.05 ‘.’ 0.1 ‘ ’ 1
> mod.emm <- emmeans(m3, ~ year)
> mod.emm
 year  emmean       SE  df asymp.LCL asymp.UCL
 a    -16.987 0.008396 Inf   -17.003   -16.970
 b      1.827 0.011868 Inf     1.804     1.850
 c      2.259 0.011867 Inf     2.236     2.282

Results are given on the log (not the response) scale.
Confidence level used: 0.95
> summary(as.glht(pairs(mod.emm)))

         Simultaneous Tests for General Linear Hypotheses

Linear Hypotheses:
            Estimate Std. Error  z value Pr(>|z|)
a - b == 0 -18.813594   0.008394 -2241.22   <2e-16 ***
a - c == 0 -19.245705   0.008394 -2292.82   <2e-16 ***
b - c == 0  -0.432111   0.011863   -36.43   <2e-16 ***
---
Signif. codes:  0 ‘***’ 0.001 ‘**’ 0.01 ‘*’ 0.05 ‘.’ 0.1 ‘ ’ 1
(Adjusted p values reported -- single-step method)

> multcomp::cld(mod.emm, alpha = 0.05, Letters = LETTERS) ##agrega las letras al
poshoc
 year  emmean       SE  df asymp.LCL asymp.UCL .group
 a    -16.987 0.008396 Inf   -17.003   -16.970  A
 b      1.827 0.011868 Inf     1.804     1.850   B
 c      2.259 0.011867 Inf     2.236     2.282    C
```

```
Results are given on the log (not the response) scale.
Confidence level used: 0.95
P value adjustment: tukey method for comparing a family of 3 estimates
significance level used: alpha = 0.05
```

Response: **Aphid**

```
      Chisq Df Pr(>Chisq)
year 15.437  2  0.0004445 ***
---
Signif. codes:  0 ‘***’ 0.001 ‘**’ 0.01 ‘*’ 0.05 ‘.’ 0.1 ‘ ’ 1
> mod.emm <- emmeans(m3, ~ year)
> mod.emm
 year emmean    SE  df asymp.LCL asymp.UCL
 a      1.04 0.649 Inf    -0.228      2.31
 b      1.96 0.604 Inf     0.772      3.14
 c      1.53 0.605 Inf     0.347      2.72

Results are given on the log (not the response) scale.
Confidence level used: 0.95
> summary(as.glht(pairs(mod.emm)))

         Simultaneous Tests for General Linear Hypotheses

Linear Hypotheses:
           Estimate Std. Error z value Pr(>|z|)
a - b == 0  -0.9128     0.2770  -3.295  0.00264 **
a - c == 0  -0.4896     0.2933  -1.669  0.20746
b - c == 0   0.4232     0.1572   2.691  0.01816 *
---
Signif. codes:  0 ‘***’ 0.001 ‘**’ 0.01 ‘*’ 0.05 ‘.’ 0.1 ‘ ’ 1
```

(Adjusted p values reported -- single-step method)

```
> multcomp::cld(mod.emm, alpha = 0.05, Letters = LETTERS) ##agrega las letras al
poshoc
 year emmean    SE  df asymp.LCL asymp.UCL .group
 a      1.04 0.649 Inf    -0.228      2.31  A
 c      1.53 0.605 Inf     0.347      2.72  A
 b      1.96 0.604 Inf     0.772      3.14   B

Results are given on the log (not the response) scale.
Confidence level used: 0.95
P value adjustment: tukey method for comparing a family of 3 estimates
significance level used: alpha = 0.05
```

Response: **L. striatellus**

```
     Chisq Df Pr(>Chisq)
year 9.6093  2   0.008192 **
---
Signif. codes:  0 ‘***’ 0.001 ‘**’ 0.01 ‘*’ 0.05 ‘.’ 0.1 ‘ ’ 1
> mod.emm <- emmeans(m3, ~ year)
> mod.emm
 year emmean    SE  df asymp.LCL asymp.UCL
 a      2.35 0.380 Inf      1.61       3.1
 b      2.33 0.291 Inf      1.76       2.9
 c      1.83 0.292 Inf      1.25       2.4

Results are given on the log (not the response) scale.
Confidence level used: 0.95
> summary(as.glht(pairs(mod.emm)))
```

```
	Simultaneous Tests for General Linear Hypotheses

Linear Hypotheses:
           Estimate Std. Error z value Pr(>|z|)
a - b == 0  0.01875    0.30639   0.061  0.99786
a - c == 0  0.52559    0.31516   1.668  0.20831
b - c == 0  0.50684    0.16849   3.008  0.00686 **
---
Signif. codes:  0 ‘***’ 0.001 ‘**’ 0.01 ‘*’ 0.05 ‘.’ 0.1 ‘ ’ 1
(Adjusted p values reported -- single-step method)

> multcomp::cld(mod.emm, alpha = 0.05, Letters = LETTERS) ##agrega las letras al poshoc
 year emmean    SE  df asymp.LCL asymp.UCL .group
 c      1.83 0.292 Inf      1.25       2.4  A
 b      2.33 0.291 Inf      1.76       2.9   B
 a      2.35 0.380 Inf      1.61       3.1  AB

Results are given on the log (not the response) scale.
Confidence level used: 0.95
P value adjustment: tukey method for comparing a family of 3 estimates
significance level used: alpha = 0.05
```

Response: **Orius sp.**

```
      Chisq Df Pr(>Chisq)
year 40.548  2  1.567e-09 ***
---
Signif. codes:  0 ‘***’ 0.001 ‘**’ 0.01 ‘*’ 0.05 ‘.’ 0.1 ‘ ’ 1
> mod.emm <- emmeans(m3, ~ year)
> mod.emm
```

```
 year emmean    SE  df asymp.LCL asymp.UCL
 a     0.809 0.373 Inf    0.0775      1.54
 b     2.527 0.285 Inf    1.9681      3.09
 c     2.037 0.286 Inf    1.4768      2.60

Results are given on the log (not the response) scale.
Confidence level used: 0.95
> summary(as.glht(pairs(mod.emm)))

	 Simultaneous Tests for General Linear Hypotheses

Linear Hypotheses:
           Estimate Std. Error z value Pr(>|z|)
a - b == 0  -1.7179     0.2849  -6.029  < 1e-04 ***
a - c == 0  -1.2273     0.2891  -4.245  < 1e-04 ***
b - c == 0   0.4905     0.1458   3.363  0.00205 **
---
Signif. codes:  0 ‘***’ 0.001 ‘**’ 0.01 ‘*’ 0.05 ‘.’ 0.1 ‘ ’ 1
(Adjusted p values reported -- single-step method)

> multcomp::cld(mod.emm, alpha = 0.05, Letters = LETTERS) ##agrega las letras al poshoc
 year emmean    SE  df asymp.LCL asymp.UCL .group
 a     0.809 0.373 Inf    0.0775      1.54  A
 c     2.037 0.286 Inf    1.4768      2.60   B
 b     2.527 0.285 Inf    1.9681      3.09    C

Results are given on the log (not the response) scale.
Confidence level used: 0.95
P value adjustment: tukey method for comparing a family of 3 estimates
significance level used: alpha = 0.05
```

```
Response: Aeolothrips sp.
      Chisq Df Pr(>Chisq)
year 11.705  2   0.002873 **
---
Signif. codes:  0 '***' 0.001 '**' 0.01 '*' 0.05 '.' 0.1 ' ' 1
> mod.emm <- emmeans(m3, ~ year)
> mod.emm
 year emmean    SE  df asymp.LCL asymp.UCL
 a     0.584 0.708 Inf    -0.804      1.97
 b     1.861 0.612 Inf     0.662      3.06
 c     1.375 0.611 Inf     0.177      2.57

Results are given on the log (not the response) scale.
Confidence level used: 0.95
> summary(as.glht(pairs(mod.emm)))

         Simultaneous Tests for General Linear Hypotheses

Linear Hypotheses:
           Estimate Std. Error z value Pr(>|z|)
a - b == 0  -1.2769     0.4234  -3.016  0.00662 **
a - c == 0  -0.7905     0.4325  -1.828  0.15174
b - c == 0   0.4864     0.2184   2.227  0.06210 .
---
Signif. codes:  0 '***' 0.001 '**' 0.01 '*' 0.05 '.' 0.1 ' ' 1
(Adjusted p values reported -- single-step method)

> multcomp::cld(mod.emm, alpha = 0.05, Letters = LETTERS) ##agrega las letras al poshoc
 year emmean    SE  df asymp.LCL asymp.UCL .group
```

```
 a     0.584 0.708 Inf    -0.804      1.97  A
 c     1.375 0.611 Inf     0.177      2.57  AB
 b     1.861 0.612 Inf     0.662      3.06   B

Results are given on the log (not the response) scale.
Confidence level used: 0.95
P value adjustment: tukey method for comparing a family of 3 estimates
significance level used: alpha = 0.05

>
```

Response: **Coccinellidae**

```
      Chisq Df Pr(>Chisq)
year 19.512  2  5.794e-05 ***
---
Signif. codes:  0 '***' 0.001 '**' 0.01 '*' 0.05 '.' 0.1 ' ' 1
> mod.emm <- emmeans(m3, ~ year)
> mod.emm
 year emmean    SE  df asymp.LCL asymp.UCL
 a     0.706 0.354 Inf    0.0118     1.401
 b     1.034 0.234 Inf    0.5748     1.494
 c     0.160 0.243 Inf   -0.3172     0.637

Results are given on the log (not the response) scale.
Confidence level used: 0.95
> summary(as.glht(pairs(mod.emm)))

         Simultaneous Tests for General Linear Hypotheses

Linear Hypotheses:
           Estimate Std. Error z value Pr(>|z|)
a - b == 0  -0.3277     0.3348  -0.979    0.581
```

```
a - c == 0   0.5465     0.3483   1.569    0.250
b - c == 0   0.8742     0.1979   4.417   <1e-04 ***
---
Signif. codes:  0 ‘***’ 0.001 ‘**’ 0.01 ‘*’ 0.05 ‘.’ 0.1 ‘ ’ 1
(Adjusted p values reported -- single-step method)

> multcomp::cld(mod.emm, alpha = 0.05, Letters = LETTERS) ##agrega las letras al poshoc
 year emmean    SE  df asymp.LCL asymp.UCL .group
 c     0.160 0.243 Inf   -0.3172     0.637  A
 a     0.706 0.354 Inf    0.0118     1.401  AB
 b     1.034 0.234 Inf    0.5748     1.494   B

Results are given on the log (not the response) scale.
Confidence level used: 0.95
P value adjustment: tukey method for comparing a family of 3 estimates
significance level used: alpha = 0.05
```
